# Poor replication validity of biomedical association studies reported by newspapers

**DOI:** 10.1371/journal.pone.0172650

**Published:** 2017-02-21

**Authors:** Estelle Dumas-Mallet, Andy Smith, Thomas Boraud, François Gonon

**Affiliations:** 1 Centre Emile Durkheim, CNRS UMR5116 at Université de Bordeaux, Pessac, France; 2 Institute of Neurodegenerative Diseases, CNRS UMR5293 at Université de Bordeaux, Bordeaux, France; Tilburg University, NETHERLANDS

## Abstract

**Objective:**

To investigate the replication validity of biomedical association studies covered by newspapers.

**Methods:**

We used a database of 4723 primary studies included in 306 meta-analysis articles. These studies associated a risk factor with a disease in three biomedical domains, psychiatry, neurology and four somatic diseases. They were classified into a lifestyle category (e.g. smoking) and a non-lifestyle category (e.g. genetic risk). Using the database *Dow Jones Factiva*, we investigated the newspaper coverage of each study. Their replication validity was assessed using a comparison with their corresponding meta-analyses.

**Results:**

Among the 5029 articles of our database, 156 primary studies (of which 63 were lifestyle studies) and 5 meta-analysis articles were reported in 1561 newspaper articles. The percentage of covered studies and the number of newspaper articles per study strongly increased with the impact factor of the journal that published each scientific study. Newspapers almost equally covered initial (5/39 12.8%) and subsequent (58/600 9.7%) lifestyle studies. In contrast, initial non-lifestyle studies were covered more often (48/366 13.1%) than subsequent ones (45/3718 1.2%). Newspapers never covered initial studies reporting null findings and rarely reported subsequent null observations. Only 48.7% of the 156 studies reported by newspapers were confirmed by the corresponding meta-analyses. Initial non-lifestyle studies were less often confirmed (16/48) than subsequent ones (29/45) and than lifestyle studies (31/63). Psychiatric studies covered by newspapers were less often confirmed (10/38) than the neurological (26/41) or somatic (40/77) ones. This is correlated to an even larger coverage of initial studies in psychiatry. Whereas 234 newspaper articles covered the 35 initial studies that were later disconfirmed, only four press articles covered a subsequent null finding and mentioned the refutation of an initial claim.

**Conclusion:**

Journalists preferentially cover initial findings although they are often contradicted by meta-analyses and rarely inform the public when they are disconfirmed.

## Introduction

Over recent years many scientific editorials have highlighted the "reproducibility crisis" of biomedical findings [1–5]. Empirical studies have actually observed that many initial findings are refuted by subsequent studies, a trend that holds true at all three levels of biomedical research: i) preclinical studies [6, 7], ii) associations between biomarkers or risk factors and diseases [8–11], and iii) clinical trials [12–16]. These empirical studies, as well as theoretical considerations [1], have highlighted that initial findings on a novel scientific question are especially vulnerable to refutation. However, others have argued that it is a mistake to expect 100% replication [17]: “a single study almost never provides definitive resolution for or against an effect and its explanation.” Scientific progress is a cumulative process that reduces uncertainty by combining uncertain initial findings with validation by subsequent studies. In other words, the low replication validity of many initial studies does not hinder the advancement of knowledge as long as science remains self-corrective [18].

However, an unrecognized consequence of the low replication validity of single studies concerns their media coverage. In the case of attention deficit hyperactivity disorder (ADHD), we previously found that newspapers preferentially report initial findings although most are refuted or strongly attenuated by subsequent studies [19]. We examined the replication validity of ten biomedical studies related to ADHD, which were widely reported by the media during the 1990’s. Among them, seven were found to be initial studies and all seven were either refuted or strongly attenuated by subsequent studies [19]. However, the strength of evidence provided by this preliminary study was limited by its sample size. The present study examined the preferential media coverage of early biomedical findings on a larger scale. Another weakness of our preliminary study was related to the heterogeneity of the ten ADHD studies that were examined: one was a pre-clinical study in mice, another estimated ADHD prevalence, three investigated the effectiveness of medication and five investigated the association of ADHD with biomarkers or risk factors. In the present study we did not consider pre-clinical studies because they are rarely included in meta-analyses, thus precluding assessment of their replication validity. We also excluded clinical trials investigating treatment effectiveness because their publication is influenced by stakeholders, in most cases pharmaceutical companies [20, 21], and because their media coverage is biased by this influence [22, 23]. Because this influence represents a parameter that might confound our analysis, we decided to focus on studies associating biomarkers and risk factors with diseases. This type of biomedical research is crucial for improving the understanding, diagnosis and prevention of diseases and is thus often reported by newspapers.

We recently built a large database of 595 meta-analyses investigating biomarkers and risk factors associated with four psychiatric disorders, four neurological pathologies and four somatic diseases [11]. Starting from the assumption that each meta-analysis provided the best estimate of the ‘true’ effect size, we showed that most effect sizes reported by initial studies were inflated. In the present study we investigated three questions. First, we quantified and characterized the newspaper coverage of the scientific publications included in our database. Second, we tested whether scientific observations reported by newspapers were confirmed, attenuated or refuted by the corresponding meta-analyses. We explored several factors that might influence the percentage of studies covered by newspapers and the replication validity of scientific observations that are covered: the type of study (initial versus subsequent studies and null findings versus "positive" ones), the prestige of the scientific journal that published them, the biomedical domain and the type of association. In particular, as previous studies showed that newspapers preferentially cover studies associating diseases with lifestyle risk factors (e.g. smoking) [24, 25], we considered this type of association to be a category. Third, we analyzed the possible media coverage of subsequent studies contradicting initial claims that had previously been reported by newspapers.

## Methods

### Scientific studies and their classification

The database described in our previous publication [11] included 663 meta-analyses investigating the association between biomarkers, risk factors or cognitive/behavioral observations and twelve pathologies belonging to three biomedical domains: psychiatry (Attention Deficit Hyperactivity Disorder (ADHD), autism, major depression, and schizophrenia), neurology (Alzheimer's and Parkinson's diseases, epilepsy, multiple sclerosis) and four somatic diseases (breast cancer, glaucoma, psoriasis and rheumatoid arthritis). In a preliminary step of the present study we observed that initial studies related to cognitive/behavioral observations were never covered by newspapers. Therefore, the 68 meta-analyses dealing with cognitive/behavioral observations were not considered in the present study. The 595 meta-analyses considered here were reported by 306 meta-analysis articles and included primary observations reported in 4775 scientific articles. As described previously, initial studies were distinguished from subsequent ones on the basis of their publication date (their references are given in our previous study [11]). From this database we excluded primary articles published before 1981. Indeed, we used the *Dow Jones Factiva* database to test whether these primary articles were covered by newspapers and the *Factiva* database starts on January 2nd 1981. Thus our final database includes 5029 scientific articles: 405 initial studies, 4218 subsequent studies and 306 meta-analysis articles.

In our previous study, these 595 meta-analyses were classified into three types: genetic studies, brain imaging studies and "other" studies. This latter type included a wide variety of biochemical, biophysical and epidemiological studies. Previous studies [24, 25] and a preliminary step of the present study led us to subdivide all association studies into two categories: a lifestyle category corresponding to any association between a pathology and a risk factor on which each subject can individually decide to act (e.g. red meat consumption, smoking, non compulsory vaccination) and a non-lifestyle category.

### Impact factor of the journals that published scientific studies

We used the *Journal Citation Reports* from *The Web of Science* to determine the 2012 impact factor of the journals that published the 5029 articles of our database. In our previous study we observed that studies associating ADHD with a biomarker or a risk factor were rarely covered by newspapers if they were published in a scientific journal with an impact factor below five [19]. Therefore, when testing whether each study of our database was echoed by newspapers, we limited our initial search to articles published in scientific journals with an impact factor equal or superior to five. We observed that among the 91 scientific articles reported by newspapers and related to other pathologies than breast cancer, the lowest impact factor was 6.5. We found no press coverage for the 220 articles published in journals with an impact factor between 5 and 6.5. Therefore, it seems unlikely that the 2342 primary studies not related to breast cancer and published in journals with an impact factor below five attracted journalists' attention. As a result, we did not check the media coverage of these 2342 primary studies. Regarding breast cancer, among the 64 scientific articles reported by newspapers, we found 14 articles with an impact factor between 5 and 6.5. Therefore, we extended our search for a possible media coverage of breast cancer studies with an impact factor between 4 and 5. However, we found no media coverage for these 340 articles. Therefore, we did not check the media coverage of the 339 primary studies related to breast cancer with an impact factor below four.

In order to test the influence of the impact factor on media coverage, we considered three classes. Prestigious journals with an impact factor ≥ 30 (*Nature*, *Science*, *JAMA*, *The Lancet* and *The New England Journal of Medicine*) formed the first class. The limit between the two other classes was adjusted in order to keep the number of covered articles roughly equal in both classes. Thus, the second class included articles published in journals with an impact factor between 10 and 30 and the third one all other articles (IF ≤ 10).

### Newspaper coverage of scientific studies

As previously described [19], we used the *Dow Jones Factiva* database to find newspaper articles echoing the scientific studies of our database. Briefly, the following keywords were used in each search: "study OR research* OR scientist*". Moreover, each search also used specific keywords that included the name of the pathology, one or several keywords that characterized the study (e.g. "gene", "milk", "birth weight") and was restricted to one month after the publication date of the study. Some searches, particularly about genetic associations, led to many items. In those cases, we added the name of the authors of the study, or of the university to the keywords. We only considered newspaper articles written in English and published in the general press. Articles published in the specialized press (e.g. *Pharma Business Week*), as well as articles published by any press agency (e.g. *Associated Press*), were not taken into account. Sometimes newspaper articles found by this search actually highlighted a biomedical finding that was not included in our database of 595 meta-analyses. For example, some newspaper articles underlined risk factors associated with colon cancer, but did not mention the association of the same risk factor with breast cancer although it was also reported in the corresponding scientific article. In these few cases this media coverage was not considered. This search was performed by one author (EDM). Another author (FG) independently checked the possible media coverage of 20% of scientific studies. Both authors always reached agreement on whether any scientific study was echoed or not. A few minor disagreements occurred regarding the number of newspaper articles reporting some studies and they were resolved by discussion.

### Replication validity of scientific observations reported by newspapers

We used our previously published database of 595 meta-analyses to check whether biomedical findings echoed by newspapers were confirmed, or not, by the corresponding meta-analyses. As previously described [11], a primary observation was considered in agreement with the corresponding meta-analysis if it satisfied two criteria: first, both observations were in nominal agreement (i.e. both reported a statistically significant effect in the same direction or the absence of a significant effect) and, second, when both reported a significant effect, the effect size of the primary observation must not be more than twice as large as that of the corresponding meta-analysis (i.e. effect size inflation below 100%). Sometimes single scientific articles reported several associations included in our 595 meta-analyses while newspaper articles that echoed them did not specify which association they highlighted. This is often the case with genetic studies: newspapers reported that new genes have been found to be associated with a disease, whereas the scientific article described significant associations with several single nucleotide polymorphisms. In these cases we considered the replication validity of the most credible association (i.e. with the smallest *p* value of the statistical test). Thus, we assessed the replication validity of any scientific article covered by newspapers under the most favorable conditions.

## Results

### Overview of scientific articles reported by newspapers

Among the 4723 primary studies of our database 156 were echoed by at least one newspaper article (Table 1). These 156 studies were covered by 1475 newspaper articles and were related to ten pathologies (ADHD, autism, major depression, schizophrenia, Alzheimer's and Parkinson's diseases, multiple sclerosis, breast cancer, glaucoma and rheumatoid arthritis). The primary studies included in our database and related to epilepsy or psoriasis did not attract newspaper attention. Moreover, 86 newspaper articles covered five out of the 306 meta-analysis articles of our database. The references of these 156 studies and of the corresponding meta-analysis articles are given in Supporting Information (S1 Text). The raw data about the studies covered by newspapers and their corresponding meta-analyses are also given (S1 File).

**Table 1:** Number of primary articles included in meta-analyses and number of primary articles covered by newspapers classified by domains and association types.

Primary articles	All	lifestyle	non-lifestyle
PSY	1905	63	1842
NEURO	1279	158	1121
SOMA	1539	418	1121
All	4723	639	4084
*Covered articles*			
PSY	38 (1.99)	6 (9.52)	32 (1.74)
NEURO	41 (3.21)	13 (8.23)	28 (2.50)
SOMA	77 (5.0)	44 (10.53)	33 (2.94)
All	156 (3.30)	63 (9.86)	93 (2.28)
**Meta-analysis articles**			
all	306	37	269
covered	5 (1.63)	2 (5.41)	3 (1.11)

PSY: psychiatry, NEURO: Neurology, SOMA: somatic diseases, lifestyle: association between a pathology and a risk factor on which each subject can act, non-lifestyle: any other association studies. The percentage of articles covered by newspapers is indicated in parentheses.

The number of newspaper articles echoing each of these 161 scientific articles (156 primary and 5 meta-analysis articles) ranged from 1 to 50 and this number was positively correlated with the impact factor of the journal that published them (linear regression, R^2^ = 0.107, F-test on variance equality: p = 0.003, Pearson's test: p < 0.0001).

### Biomedical domain and association type of covered studies

The percentage of primary articles covered by newspapers ranged from 1.74% to 10.5% depending on the biomedical domain and the association type (Table 1). Whatever the domain, lifestyle studies were more often covered than non-lifestyle ones (Table 1). Compared to this major effect of association type, the coverage rate does not strongly differ between biomedical domains (Table 1). Newspapers also covered a larger proportion of meta-analysis articles of the lifestyle category (Table 1). Altogether, newspapers echoed 161 scientific articles, of which 65 (40.4%) were of the lifestyle category. Moreover, lifestyle associations tended to receive a larger newspaper coverage (mean: 11.9 newspaper articles per study) than non-lifestyle associations (mean: 8.2 newspaper articles per study). However, this difference does not reach statistical significance. Overall, this larger press coverage of lifestyle studies was not related to the impact factor of the scientific journals that published them. Indeed, lifestyle scientific studies were published in less prestigious journals (median IF = 10.6) than those of the non-lifestyle category (median IF = 17.6) and this difference is statistically significant (unpaired t test, p = 0.0003).

### Media coverage of initial studies versus subsequent ones

**Fig 1:**
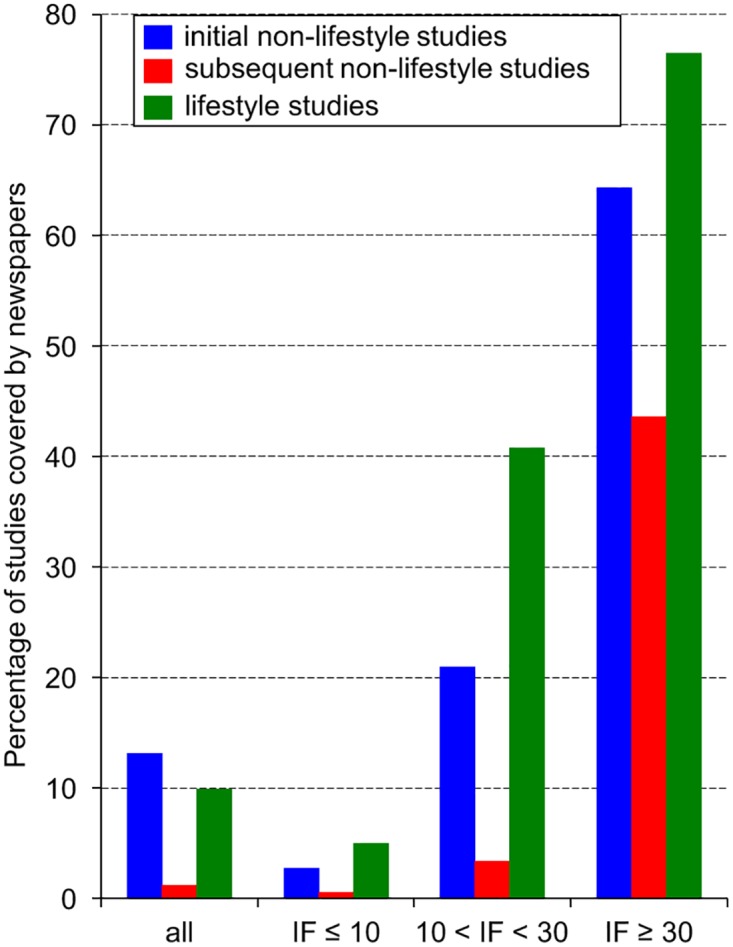
Preferential coverage of initial findings and influence of the impact factor (IF). The figure shows the percentage of primary studies that are covered by newspapers depending on the study type (lifestyle versus non-lifestyle). Studies of the lifestyle category described associations linking a pathology to a risk factor on which each subject can act. Regarding non-lifestyle articles, the figure also contrasts initial articles with subsequent ones. Differences in the media coverage between initial studies and subsequent ones were statistically significant (see text) except for studies published in prestigious journals (IF ≥ 30). Raw data are given in Supporting Information (S2 Text).

Initial studies attract more media attention than subsequent studies. Indeed, 13.1% of initial studies were covered by newspapers whereas only 2.4% of subsequent studies were (raw data are given in Supporting Information: S2 Text). This preferential coverage is largely influenced by the type of association and the impact factor of the scientific journal. Among the 639 primary articles of the lifestyle category, 39 reported initial findings and 600 subsequent observations. Newspapers almost equally covered these initial findings (5 covered articles, 12.8%) and these subsequent observations (58 covered articles, 9.7%). In contrast, among the 4084 primary articles investigating non-lifestyle associations, newspapers preferentially covered initial findings (Fig 1). Indeed, they echoed 48 of the 366 initial non-lifestyle studies (13.1%), but only 45 of the 3718 subsequent ones (1.2%). This difference reaches statistical significance (Chi2 test: X^2^ = 211.52 p<0.0001). This preferential coverage of initial non-lifestyle studies also holds true when considering articles published in scientific journals with an impact factor below 10 (Chi 2 test: X^2^ = 17.73 p<0.0001) and with an impact factor between 10 and 30 (Chi 2 test: X^2^ = 29.11 p<0.0001) (Fig 1). However, for the 83 non-lifestyle articles published in prestigious journals, no significant preferential coverage of initial observations was observed (Chi 2 test: X^2^ = 3.49 p = 0.062). Finally, the percentage of articles covered by newspapers strongly increased with the impact factor (Fig 1). This effect is statistically significant regarding initial or subsequent non-lifestyle studies (2x3 Chi 2 tests: X^2^ = 124.4 p<0.0001 or X^2^ = 612.1 p<0.0001, respectively) and for lifestyle studies (2x3 Fisher test: p<0.0001). Therefore, publishing a study in a prestigious journal considerably increases its chance of being covered by newspapers irrespective of the type of association investigated or its newness.

### Media coverage of "positive" versus null findings

All 53 initial studies covered by newspapers reported a "positive" finding (i.e. a statistically significant effect). It follows that none of the 174 initial studies included in our database and reporting a null effect (i.e. 43% of all initial studies) attracted journalists' attention (Table 2). Among the 103 subsequent studies covered by newspapers, only 14 studies reported a null effect. Regarding the non-lifestyle associations, among the 45 subsequent studies covered by newspapers, only five reported a null finding. Moreover this media coverage was small (10 articles) compared to that of all non-lifestyle studies (742 newspaper articles). The 58 subsequent studies of the lifestyle category were echoed by 733 newspaper articles. Only nine subsequent studies reported the absence of a significant association and these null findings were reported by 65 newspaper articles. Therefore, assuming that, in our database, the percentage of subsequent studies reporting a null finding is at least as large as that of initial studies (43%) we observed that newspapers under-reported them. Indeed, only 13.6% of the subsequent studies covered by newspapers reported a null finding (Table 2). Moreover, the number of newspaper articles covering each null finding was small, particularly regarding non-lifestyle studies (Table 2). Finally, only one of the five meta-analysis articles covered by newspapers reported a null finding. Moreover, this null finding was echoed by only four newspaper articles whereas the four "positive" meta-analyses attracted 82 press articles.

**Table 2:** Newspaper coverage of primary studies reporting null findings.

	all	lifestyle	non-lifestyle
**all initial studies in the database**	405	41	364
"positive" findings	231	21	210
null findings	174	20	154
(% of null findings)	(43%)	(48.8%)	(42.3%)
**initial studies covered:**			
all	53	5	48
null findings	0	0	0
**subsequent studies covered:**			
all	103	58	45
null findings	14	9	5
**Newspaper articles:**			
all	1475	733	742
covering null findings	75	65	10

### Replication validity of primary studies reported by newspapers

**Fig 2:**
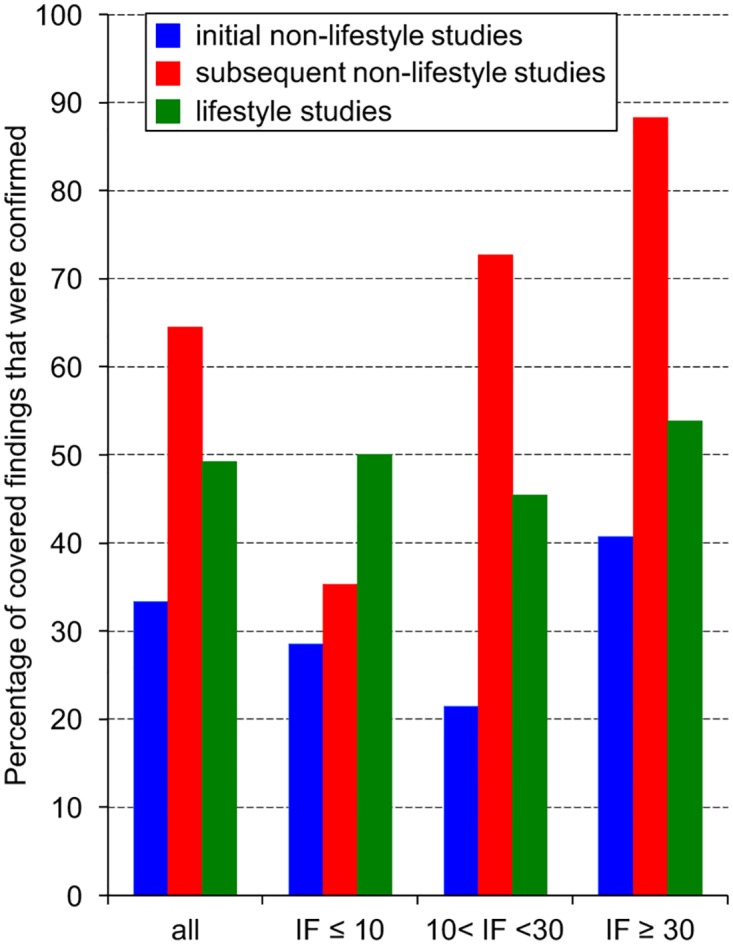
Replication validity of primary articles reported by newspapers. The figure shows the percentage of primary articles echoed by newspapers whose main finding was consistent with the corresponding meta-analysis. We considered here the same three categories as in Fig 1: primary articles of the lifestyle category and initial or subsequent non-lifestyle studies. Raw data are given in Supporting Information (S2 Text).

Overall, among the 156 primary studies echoed by newspapers 76 (48.7%) reported a main finding consistent with the corresponding meta-analysis (raw data are given in Supporting Information: S2 Text). Among the 156 primary studies covered by newspapers 53 were initial studies of which only 18 (34.0%) were confirmed by the corresponding meta-analyses. In contrast, of the 103 subsequent studies 58 (56.3%) were confirmed and this difference is statistically significant (Chi2 test: X^2^ = 6.99 p = 0.0082). Regarding the 63 lifestyle studies echoed by newspapers, 49.2% of their findings were confirmed by the corresponding meta-analyses without any effect of the impact factor (Fig 2). Only 33.3% of the main findings reported by the 48 initial non-lifestyle studies and covered by newspapers were actually validated by the corresponding meta-analyses. This replication rate was not influenced by the impact factor (Fig 2). In contrast the 45 subsequent non-lifestyle studies covered by newspapers were more often consistent with the corresponding meta-analyses than the initial non-lifestyle studies (Fig 2). Their replication rate was indeed larger than that of initial studies whether considering them as a whole (64.4% versus 33.3% for initial studies; Chi2 test, X^2^ = 9.0 p = 0.0027) or when considering only the 17 studies published in prestigious journals (88.2% versus 40.7% for initial studies; Fisher test, p = 0.0021). The replication rate of subsequent non-lifestyle studies increased with the impact factor (2x3 Fisher test, p = 0.0055).

**Fig 3:**
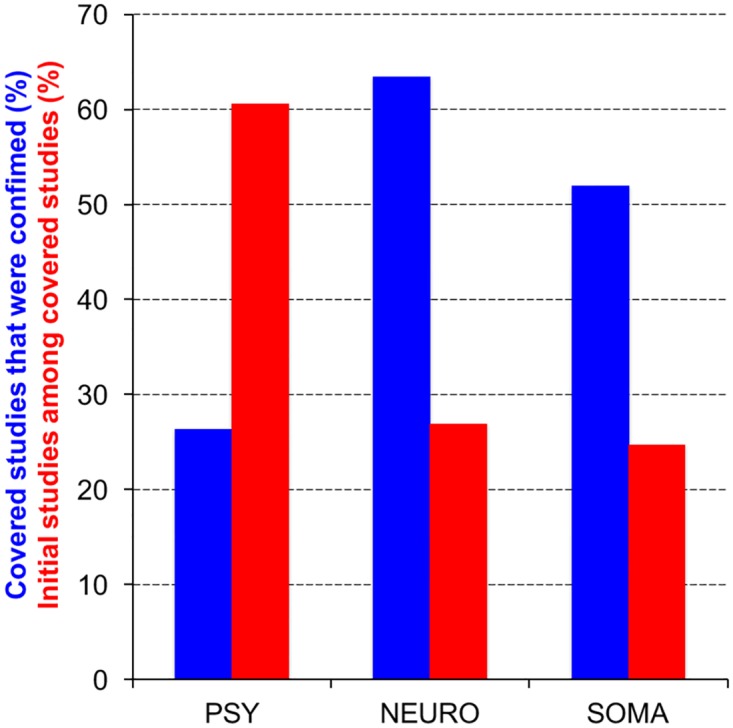
Replication validity of primary studies reported by newspapers in three biomedical domains. The blue bars show the percentage of primary studies covered by newspapers whose main finding was consistent with the corresponding meta-analysis. The red bars show the percentage of initial findings among primary studies echoed by newspapers and related to four psychiatric disorders (PSY), four neurological diseases (NEURO) and four somatic diseases (SOMA). Raw data are given in Supporting Information (S2 Text).

When we classified the 156 primary studies echoed by newspapers according to the biomedical domain (Fig 3), studies related to psychiatric disorders were less often confirmed by the corresponding meta-analyses than those related to neurological diseases (chi2 test; X^2^ = 10.94 p = 0.0009) and to somatic diseases (X^2^ = 6.8 p = 0.0091). Moreover, studies related to psychiatry and covered by newspapers were more often initial findings than those in neurology (X^2^ = 9.13 p = 0.0025) and for somatic diseases (X^2^ = 14.11 p = 0.0002). The low replication validity of studies in psychiatry appears thus to be correlated with the fact that newspapers favor initial findings in this domain even more than in other domains. This correlation further supports our previous conclusion [19]: the replication validity of biomedical studies covered by newspapers is poor because journalists favor initial findings over subsequent studies.

### Analysis of primary studies covered by three newspapers or more

When considering only the 102 primary studies covered by at least three newspaper articles, we observed the same patterns (raw data are given in Supporting Information: S2 Text). Journalists covered initial studies more often (32/405) than subsequent studies (70/4318). In particular, non-lifestyle studies were ten times more often echoed when they reported initial findings than subsequent observations. Of these 102 primary studies, 53 (52.0%) were confirmed by the corresponding meta-analyses. The replication validity of the 28 subsequent non-lifestyle observations was still higher than that of the 29 initial findings (71.4% versus 41.4%; Chi2 test, X^2^ = 5.22 p = 0.022). Likewise, studies related to psychiatry and covered by newspapers were more often initial findings than those in neurology (Fisher test; p = 0.0072) and for somatic diseases (Chi2 test X^2^ = 9.59 p = 0.002). They were also confirmed less often (30.4%) than those related to neurological diseases (64%) (chi2 test; X^2^ = 5.41 p = 0.020) and to somatic diseases (55.6) (X^2^ = 4.08 p = 0.043).

### Lack of media coverage of studies contradicting initial claims

Among the 53 initial studies covered by newspapers, 35 (66%) were disconfirmed by the corresponding meta-analyses. These 35 initial studies were followed by 503 subsequent studies of which 398 reported either the absence of a statistically significant effect or a significant effect in the opposite direction. Only one of these 398 subsequent studies and only one of the 35 corresponding meta-analysis articles were covered by newspapers. In the first case, the initial study published in 1999 by *The Lancet* (IF = 39) reported that the dopamine transporter level was increased (+70%) in both striatum of ADHD patients [26]. This initial study was covered by 22 newspaper articles. In contrast, according to a subsequent study published in 2009 by *JAMA* (IF = 30), the dopamine transporter level was significantly decreased (-19%) in the left striatum of ADHD patients, but not in the right [27]. The three newspapers that covered this subsequent study did not mention that it contradicted the initial claim although two newspapers (*The Globe and Mail* and *The Washington Post*) had also covered the initial study.

In the second case, the initial study was published in 2003 in *Science* and claimed that a genetic factor was associated with depression when subjects were exposed to stressful life events [28]. This finding was widely covered by newspapers (50 articles). Two subsequent studies that confirmed the same association were also echoed (7 and 2 newspaper articles). Newspapers never covered the eleven subsequent studies that failed to replicate this genetic association. Finally, the meta-analysis published in 2009 by Risch and coworkers in *JAMA* (IF = 30) and contradicting "positive" findings [29], was covered by only four newspaper articles and all four mentioned that the initial finding "has not held up to scientific scrutiny". Overall, 234 newspapers articles covered the 35 initial studies that were later disconfirmed by subsequent meta-analyses. However, only four newspaper articles explicitly mentioned the refutation of an initial claim previously covered in the press.

## Comments

We explored the replication validity of association studies echoed by the lay press. Altogether, 48.7% of the studies examined were confirmed by subsequent meta-analyses. However, subpopulation analyses show large variations of this replication rate. Newspapers preferentially echo initial studies over subsequent studies investigating the same question and initial findings covered by newspapers are less often validated by replications (34%) than subsequent findings (56.3%). This observation is consistent with several observational studies reporting that many initial or early biomedical findings are disconfirmed by subsequent studies [6–16]. Thus, the present study further supports our previous conclusion [19]: a major factor that explains the poor replication validity of biomedical studies covered by newspapers is that journalists favor initial findings over replication studies.

In agreement with previous studies [24, 25], we observed that newspapers preferentially reported lifestyle association studies linking a pathology to a risk factor on which each reader can act. Non-lifestyle studies related to brain imaging, genetic factor or other inescapable risk factors were less often echoed. This preferential coverage further supports the view that the first journalists' aim is to attract readers' attention. We also observed that newspapers never covered initial findings reporting null findings and under-reported subsequent null findings, particularly regarding non-lifestyle studies. As a consequence, journalists rarely cover null findings that contradict previously echoed studies. Within our database we observed only one interesting exception to this general rule: four newspaper articles covered the meta-analysis by Risch and coworkers and informed the public that it disconfirmed Caspi's initial study, which received much larger coverage (50 articles). All these observations support the conclusion of a case study: "scientific uncertainty *per se* is not attractive to journalists" (p. 344) [30].

We observed that findings reported by newspapers and related to psychiatric disorders were less often confirmed by meta-analyses than those related to neurological or somatic diseases. We correlated this observation with the fact that the preferential coverage of initial studies is even stronger regarding psychiatry. However, another factor might contribute to the low replication validity of psychiatric studies covered by newspapers: we previously showed that initial association studies linking biomarkers or risk factors to psychiatric disorders were less often confirmed by corresponding meta-analyses than those related to neurological diseases [11]. Likewise, highly cited early studies investigating the effectiveness of psychiatric treatments are often contradicted by subsequent studies [16].

## Limitations

Although single primary studies have often investigated several associations linking a biomarkers or risk factors to a pathology (e.g. several polymorphisms on the same gene), we restricted our replication analysis to only one association per scientific article. Because we selected the most credible association (i.e. with the highest degree of statistical significance) it is likely that the replication rates we reported here are overestimated.

The 4723 primary articles of our database were included in meta-analyses thus enabling us to assess their replication validity. Whether the newspaper coverage of association studies included in meta-analyses is representative of the coverage of all association studies remains an open question.

We only considered newspaper articles published within a month following the publication of each scientific study. Indeed, when newspaper articles were published much later, it was often impossible to identify the scientific study they were reporting on with certainty. Consequently, we did not investigate the long-term impact of scientific publications in the lay press, although there is no doubt that certain scientific studies did have such an impact. For example, Caspi's study published in 2003 about a genetic susceptibility to major depression [28] was still cited in the lay press well after its refutation in 2009 by a meta-analysis [29]. However, in relative terms, it is likely that the scientific publications with the strongest long-term impact also received the widest newspaper coverage shortly following their publication.

Our data support three conclusions. First, newspapers preferentially cover lifestyle associations over non-lifestyle ones. Second, they strongly favor initial findings over subsequent studies when they cover non-lifestyle associations, but they equally cover initial and subsequent lifestyle studies. Third, among non-lifestyle associations covered by newspapers, initial findings are less often confirmed by meta-analyses than subsequent studies. However, our number of observations is too small to know whether this third conclusion also holds true regarding lifestyle studies. Indeed, our database included only five initial lifestyle studies covered by newspapers.

We only studied newspaper articles but ignored television reporting, and this although television is the main source of information on medical sciences for Europeans (European Commission, 2007). Indeed, television programs rarely explicitly report on specific scientific publications [31]. Nevertheless, newspaper articles might indirectly influence the content of television programs by raising the attention of their producers.

## Conclusion

Biomedical researches mature from early studies that are highly uncertain to scientific consensuses built on subsequent studies and quantified by meta-analyses. Indeed, recent meta-researches have consistently shown that most early studies report inflated effect size or false positive effects [6–16]. Our study shows that many biomedical findings reported by newspapers are disconfirmed by subsequent studies. This is partly due to the fact that newspapers preferentially cover "positive" initial studies rather than subsequent observations, in particular those reporting null findings. Our study also suggests that most journalists from the general press do not know or prefer not to deal with the high degree of uncertainty inherent in early biomedical studies. Importantly, such biased newspaper coverage can have important social consequences [32, 33]. For example, a content analysis of newspaper articles covering Caspi's study showed that they emphasized the genetic side of the gene-by-environment interactions and this discourse deflected public attention away from the considerable impact of social inequalities upon health [34]. Therefore, with Susan Watts [35], we advocate that society "needs science journalism to weigh up the values and the vices of new science" (p. 151). In particular, when preparing a report on a scientific study, journalists should always ask scientists whether it is an initial finding and, if so, they should inform the public that this discovery is still tentative and must be validated by subsequent studies. Larsson and coworkers (2003) have identified the obstacles science journalists meet to accurately cope with uncertainty [36]. In particular, most interviewed journalists feel that it is difficult to find scientists who are independent from authors and who are willing to assist them. Thus, scientists, either as independent experts or as authors, are also responsible for improving the informative value of biomedical reporting in the mass media. In particular, they are responsible for the accuracy of the press releases covering their work and published by scientific editors or universities. This is not only a moral duty but also in the interest of science. Indeed, contrary to common beliefs, reception studies show that scientists are viewed by the public as more trustworthy when news coverage of health research acknowledges its uncertainty, especially if this is attributed to scientific authors [37, 38].

## Supporting information

S1 Text References of the 156 studies reported by newspapers and of their corresponding meta-analysis articles. (DOCX)

S2 Text Raw data shown in Figs 1–3 and about primary studies covered by three newspapers or more. (DOCX)

S1 File Raw data about the 156 studies covered by newspapers and their corresponding meta-analyses. (XLSX)
