# Supplementary material for: Poor replication validity of biomedical association studies reported by newspapers
Source: PLoS One. 2017 Feb 21;12(2):e0172650. doi: 10.1371/journal.pone.0172650 (PMC5319681; doi:10.1371/journal.pone.0172650)
Supplement: S1 Text — (DOCX) [file pone.0172650.s001.docx]

**References of studies covered by newspapers and their corresponding meta-analysis**

**ADHD**

**Primary studies**

Comings, D. E., Comings, B. G., Muhleman, D., Dietz, G., Shahbahrami, B., Tast, D., . . . et al. (1991). The dopamine D2 receptor locus as a modifying gene in neuropsychiatric disorders. *JAMA, 266*(13), 1793-1800.

Cook, E. H., Jr., Stein, M. A., Krasowski, M. D., Cox, N. J., Olkon, D. M., Kieffer, J. E., & Leventhal, B. L. (1995). Association of attention-deficit disorder and the dopamine transporter gene. *Am J Hum Genet, 56*(4), 993-998.

Dougherty, D. D., Bonab, A. A., Spencer, T. J., Rauch, S. L., Madras, B. K., & Fischman, A. J. (1999). Dopamine transporter density in patients with attention deficit hyperactivity disorder. *Lancet., 354*(9196), 2132-2133.

LaHoste, G. J., Swanson, J. M., Wigal, S. B., Glabe, C., Wigal, T., King, N., & Kennedy, J. L. (1996). Dopamine D4 receptor gene polymorphism is associated with attention deficit hyperactivity disorder. *Mol Psychiatry, 1*(2), 121-124.

McCann, D., Barrett, A., Cooper, A., Crumpler, D., Dalen, L., Grimshaw, K., . . . Stevenson, J. (2007). Food additives and hyperactive behaviour in 3-year-old and 8/9-year-old children in the community: a randomised, double-blinded, placebo-controlled trial. *Lancet, 370*(9598), 1560-1567. doi: 10.1016/s0140-6736(07)61306-3

Volkow, N. D., Wang, G. J., Kollins, S. H., Wigal, T. L., Newcorn, J. H., Telang, F., . . . Swanson, J. M. (2009). Evaluating dopamine reward pathway in ADHD: clinical implications. *JAMA, 302*(10), 1084-1091.

**Meta-analysis**

Fusar-Poli, P., Rubia, K., Rossi, G., Sartori, G., & Balottin, U. (2012). Striatal dopamine transporter alterations in ADHD: pathophysiology or adaptation to psychostimulants? A meta-analysis. *Am J Psychiatry, 169*(3), 264-272. doi: 10.1176/appi.ajp.2011.11060940

Gizer, I. R., Ficks, C., & Waldman, I. D. (2009). Candidate gene studies of ADHD: a meta-analytic review. *Hum Genet, 126*(1), 51-90. doi: 10.1007/s00439-009-0694-x

Nigg, J. T., Lewis, K., Edinger, T., & Falk, M. (2012). Meta-analysis of attention-deficit/hyperactivity disorder or attention-deficit/hyperactivity disorder symptoms, restriction diet, and synthetic food color additives. *J Am Acad Child Adolesc Psychiatry, 51*(1), 86-97 e88. doi: 10.1016/j.jaac.2011.10.015

Smith, T. F. (2010). Meta-analysis of the heterogeneity in association of DRD4 7-repeat allele and AD/HD: stronger association with AD/HD combined type. *Am J Med Genet B Neuropsychiatr Genet, 153B*(6), 1189-1199. doi: 10.1002/ajmg.b.31090

Wu, J., Xiao, H., Sun, H., Zou, L., & Zhu, L. Q. (2012). Role of dopamine receptors in ADHD: a systematic meta-analysis. *Mol Neurobiol, 45*(3), 605-620. doi: 10.1007/s12035-012-8278-5

**Autism**

**Primary studies**

Cook, E. H., Jr., Courchesne, R., Lord, C., Cox, N. J., Yan, S., Lincoln, A., . . . Leventhal, B. L. (1997). Evidence of linkage between the serotonin transporter and autistic disorder. *Mol Psychiatry, 2*(3), 247-250.

Courchesne, E., Yeung-Courchesne, R., Press, G. A., Hesselink, J. R., & Jernigan, T. L. (1988). Hypoplasia of cerebellar vermal lobules VI and VII in autism. *N Engl J Med, 318*(21), 1349-1354. doi: 10.1056/nejm198805263182102

Glasson, E. J., Bower, C., Petterson, B., de Klerk, N., Chaney, G., & Hallmayer, J. F. (2004). Perinatal factors and the development of autism: a population study. *Arch Gen Psychiatry, 61*(6), 618-627. doi: 10.1001/archpsyc.61.6.618

James, S. J., Cutler, P., Melnyk, S., Jernigan, S., Janak, L., Gaylor, D. W., & Neubrander, J. A. (2004). Metabolic biomarkers of increased oxidative stress and impaired methylation capacity in children with autism. *Am J Clin Nutr, 80*(6), 1611-1617.

Reichenberg, A., Gross, R., Weiser, M., Bresnahan, M., Silverman, J., Harlap, S., . . . Susser, E. (2006). Advancing paternal age and autism. *Arch Gen Psychiatry, 63*(9), 1026-1032. doi: 10.1001/archpsyc.63.9.1026

Sparks, B. F., Friedman, S. D., Shaw, D. W., Aylward, E. H., Echelard, D., Artru, A. A., . . . Dager, S. R. (2002). Brain structural abnormalities in young children with autism spectrum disorder. *Neurology, 59*(2), 184-192.

**Meta-analysis**

Frustaci, A., Neri, M., Cesario, A., Adams, J. B., Domenici, E., Dalla Bernardina, B., & Bonassi, S. (2012). Oxidative stress-related biomarkers in autism: systematic review and meta-analyses. *Free Radic Biol Med, 52*(10), 2128-2141. doi: 10.1016/j.freeradbiomed.2012.03.011

Huang, C. H., & Santangelo, S. L. (2008). Autism and serotonin transporter gene polymorphisms: a systematic review and meta-analysis. *Am J Med Genet B Neuropsychiatr Genet, 147B*(6), 903-913. doi: 10.1002/ajmg.b.30720

Hultman, C. M., Sandin, S., Levine, S. Z., Lichtenstein, P., & Reichenberg, A. (2011). Advancing paternal age and risk of autism: new evidence from a population-based study and a meta-analysis of epidemiological studies. *Mol Psychiatry, 16*(12), 1203-1212. doi: 10.1038/mp.2010.121

Stanfield, A. C., McIntosh, A. M., Spencer, M. D., Philip, R., Gaur, S., & Lawrie, S. M. (2008). Towards a neuroanatomy of autism: a systematic review and meta-analysis of structural magnetic resonance imaging studies. *Eur Psychiatry, 23*(4), 289-299. doi: 10.1016/j.eurpsy.2007.05.006

**Major Depressive Disorder**

**Primary studies**

Almeida, O. P., McCaul, K., Hankey, G. J., Norman, P., Jamrozik, K., & Flicker, L. (2008). Homocysteine and depression in later life. *Arch Gen Psychiatry, 65*(11), 1286-1294. doi: 10.1001/archpsyc.65.11.1286

Caspi, A., Sugden, K., Moffitt, T. E., Taylor, A., Craig, I. W., Harrington, H., . . . Poulton, R. (2003). Influence of life stress on depression: moderation by a polymorphism in the 5-HTT gene. *Science., 301*(5631), 386-389.

Collier, D. A., Stober, G., Li, T., Heils, A., Catalano, M., Di Bella, D., . . . Lesch, K. P. (1996). A novel functional polymorphism within the promoter of the serotonin transporter gene: possible role in susceptibility to affective disorders. *Mol Psychiatry, 1*(6), 453-460.

Colman, I., Ploubidis, G. B., Wadsworth, M. E., Jones, P. B., & Croudace, T. J. (2007). A longitudinal typology of symptoms of depression and anxiety over the life course. *Biol Psychiatry, 62*(11), 1265-1271. doi: 10.1016/j.biopsych.2007.05.012

Drevets, W. C., Price, J. L., Simpson, J. R., Jr., Todd, R. D., Reich, T., Vannier, M., & Raichle, M. E. (1997). Subgenual prefrontal cortex abnormalities in mood disorders. *Nature, 386*(6627), 824-827. doi: 10.1038/386824a0

Gale, C. R., & Martyn, C. N. (2004). Birth weight and later risk of depression in a national birth cohort. *Br J Psychiatry, 184*, 28-33.

Hariri, A. R., Mattay, V. S., Tessitore, A., Kolachana, B., Fera, F., Goldman, D., . . . Weinberger, D. R. (2002). Serotonin transporter genetic variation and the response of the human amygdala. *Science, 297*(5580), 400-403. doi: 10.1126/science.1071829

Patton, G. C., Coffey, C., Carlin, J. B., Olsson, C. A., & Morley, R. (2004). Prematurity at birth and adolescent depressive disorder. *Br J Psychiatry, 184*, 446-447.

Taylor, S. E., Way, B. M., Welch, W. T., Hilmert, C. J., Lehman, B. J., & Eisenberger, N. I. (2006). Early family environment, current adversity, the serotonin transporter promoter polymorphism, and depressive symptomatology. *Biol Psychiatry, 60*(7), 671-676. doi: 10.1016/j.biopsych.2006.04.019

Wiles, N. J., Peters, T. J., Leon, D. A., & Lewis, G. (2005). Birth weight and psychological distress at age 45-51 years: results from the Aberdeen Children of the 1950s cohort study. *Br J Psychiatry, 187*, 21-28. doi: 10.1192/bjp.187.1.21

Wilhelm, K., Mitchell, P. B., Niven, H., Finch, A., Wedgwood, L., Scimone, A., . . . Schofield, P. R. (2006). Life events, first depression onset and the serotonin transporter gene. *Br J Psychiatry, 188*, 210-215. doi: 10.1192/bjp.bp.105.009522

**Meta-analysis**

Almeida, O. P., McCaul, K., Hankey, G. J., Norman, P., Jamrozik, K., & Flicker, L. (2008). Homocysteine and depression in later life. *Arch Gen Psychiatry, 65*(11), 1286-1294. doi: 10.1001/archpsyc.65.11.1286

Arnone, D., McIntosh, A. M., Ebmeier, K. P., Munafo, M. R., & Anderson, I. M. (2012). Magnetic resonance imaging studies in unipolar depression: systematic review and meta-regression analyses. *Eur Neuropsychopharmacol, 22*(1), 1-16. doi: 10.1016/j.euroneuro.2011.05.003

Clarke, H., Flint, J., Attwood, A. S., & Munafo, M. R. (2010). Association of the 5- HTTLPR genotype and unipolar depression: a meta-analysis. *Psychol Med, 40*(11), 1767-1778. doi: 10.1017/s0033291710000516

Munafo, M. R., Brown, S. M., & Hariri, A. R. (2008). Serotonin transporter (5-HTTLPR) genotype and amygdala activation: a meta-analysis. *Biol Psychiatry, 63*(9), 852-857. doi: 10.1016/j.biopsych.2007.08.016

Paulson, J. F., & Bazemore, S. D. (2010). Prenatal and postpartum depression in fathers and its association with maternal depression: a meta-analysis. *JAMA, 303*(19), 1961-1969. doi: 10.1001/jama.2010.605

Risch, N., Herrell, R., Lehner, T., Liang, K. Y., Eaves, L., Hoh, J., . . . Merikangas, K. R. (2009). Interaction between the serotonin transporter gene (5-HTTLPR), stressful life events, and risk of depression: a meta-analysis. *JAMA, 301*(23), 2462-2471. doi: 10.1001/jama.2009.878

Wojcik, W., Lee, W., Colman, I., Hardy, R., & Hotopf, M. (2013). Foetal origins of depression? A systematic review and meta-analysis of low birth weight and later depression. *Psychol Med, 43*(1), 1-12. doi: 10.1017/s0033291712000682

**Schizophrenia**

**Primary studies**

Arinami, T., Itokawa, M., Enguchi, H., Tagaya, H., Yano, S., Shimizu, H., . . . Toru, M. (1994). Association of dopamine D2 receptor molecular variant with schizophrenia. *Lancet, 343*(8899), 703-704.

Byrne, M., Agerbo, E., Ewald, H., Eaton, W. W., & Mortensen, P. B. (2003). Parental age and risk of schizophrenia: a case-control study. *Arch Gen Psychiatry, 60*(7), 673-678. doi: 10.1001/archpsyc.60.7.673

Chumakov, I., Blumenfeld, M., Guerassimenko, O., Cavarec, L., Palicio, M., Abderrahim, H., . . . Cohen, D. (2002). Genetic and physiological data implicating the new human gene G72 and the gene for D-amino acid oxidase in schizophrenia. *Proc Natl Acad Sci U S A, 99*(21), 13675-13680. doi: 10.1073/pnas.182412499

Fannon, D., Chitnis, X., Doku, V., Tennakoon, L., O'Ceallaigh, S., Soni, W., . . . Sharma, T. (2000). Features of structural brain abnormality detected in first-episode psychosis. *Am J Psychiatry, 157*(11), 1829-1834. doi: 10.1176/appi.ajp.157.11.1829

Gerber, D. J., Hall, D., Miyakawa, T., Demars, S., Gogos, J. A., Karayiorgou, M., & Tonegawa, S. (2003). Evidence for association of schizophrenia with genetic variation in the 8p21.3 gene, PPP3CC, encoding the calcineurin gamma subunit. *Proc Natl Acad Sci U S A, 100*(15), 8993-8998. doi: 10.1073/pnas.1432927100

Lo, W. S., Lau, C. F., Xuan, Z., Chan, C. F., Feng, G. Y., He, L., . . . Xue, H. (2004). Association of SNPs and haplotypes in GABAA receptor beta2 gene with schizophrenia. *Mol Psychiatry, 9*(6), 603-608. doi: 10.1038/sj.mp.4001461

Malaspina, D., Harlap, S., Fennig, S., Heiman, D., Nahon, D., Feldman, D., & Susser, E. S. (2001). Advancing paternal age and the risk of schizophrenia. *Arch Gen Psychiatry, 58*(4), 361-367.

O'Donovan, M. C., Craddock, N., Norton, N., Williams, H., Peirce, T., Moskvina, V., . . . Cloninger, C. R. (2008). Identification of loci associated with schizophrenia by genome-wide association and follow-up. *Nat Genet, 40*(9), 1053-1055. doi: 10.1038/ng.201

Shenton, M. E., Kikinis, R., Jolesz, F. A., Pollak, S. D., LeMay, M., Wible, C. G., . . . et al. (1992). Abnormalities of the left temporal lobe and thought disorder in schizophrenia. A quantitative magnetic resonance imaging study. *N Engl J Med, 327*(9), 604-612. doi: 10.1056/nejm199208273270905

Shifman, S., Bronstein, M., Sternfeld, M., Pisante-Shalom, A., Lev-Lehman, E., Weizman, A., . . . Darvasi, A. (2002). A highly significant association between a COMT haplotype and schizophrenia. *Am J Hum Genet, 71*(6), 1296-1302. doi: 10.1086/344514

Sipos, A., Rasmussen, F., Harrison, G., Tynelius, P., Lewis, G., Leon, D. A., & Gunnell, D. (2004). Paternal age and schizophrenia: a population based cohort study. *Bmj, 329*(7474), 1070. doi: 10.1136/bmj.38243.672396.55

Stefansson, H., Sigurdsson, E., Steinthorsdottir, V., Bjornsdottir, S., Sigmundsson, T., Ghosh, S., . . . Stefansson, K. (2002). Neuregulin 1 and susceptibility to schizophrenia. *Am J Hum Genet, 71*(4), 877-892. doi: 10.1086/342734

Straub, R. E., Jiang, Y., MacLean, C. J., Ma, Y., Webb, B. T., Myakishev, M. V., . . . Kendler, K. S. (2002). Genetic variation in the 6p22.3 gene DTNBP1, the human ortholog of the mouse dysbindin gene, is associated with schizophrenia. *Am J Hum Genet, 71*(2), 337-348. doi: 10.1086/341750

Suddath, R. L., Christison, G. W., Torrey, E. F., Casanova, M. F., & Weinberger, D. R. (1990). Anatomical abnormalities in the brains of monozygotic twins discordant for schizophrenia. *N Engl J Med, 322*(12), 789-794. doi: 10.1056/nejm199003223221201

Zhao, X., Tang, R., Gao, B., Shi, Y., Zhou, J., Guo, S., . . . He, L. (2007). Functional variants in the promoter region of Chitinase 3-like 1 (CHI3L1) and susceptibility to schizophrenia. *Am J Hum Genet, 80*(1), 12-18. doi: 10.1086/510438

**Meta-analysis**

De Peri, L., Crescini, A., Deste, G., Fusar-Poli, P., Sacchetti, E., & Vita, A. (2012). Brain structural abnormalities at the onset of schizophrenia and bipolar disorder: a meta-analysis of controlled magnetic resonance imaging studies. *Curr Pharm Des, 18*(4), 486-494.

Haijma, S. V., Van Haren, N., Cahn, W., Koolschijn, P. C., Hulshoff Pol, H. E., & Kahn, R. S. (2013). Brain volumes in schizophrenia: a meta-analysis in over 18 000 subjects. *Schizophr Bull, 39*(5), 1129-1138. doi: 10.1093/schbul/sbs118

Liu, Z. W., Liu, J. L., An, Y., Zhang, L., & Wang, Y. M. (2012). Association between Ser311Cys polymorphism in the dopamine D2 receptor gene and schizophrenia risk: a meta-analysis in Asian populations. *Genet Mol Res, 11*(1), 261-270. doi: 10.4238/2012.February.8.1

Miller, B., Messias, E., Miettunen, J., Alaraisanen, A., Jarvelin, M. R., Koponen, H., . . . Kirkpatrick, B. (2011). Meta-analysis of paternal age and schizophrenia risk in male versus female offspring. *Schizophr Bull, 37*(5), 1039-1047. doi: 10.1093/schbul/sbq011

Munafo, M. R., Attwood, A. S., & Flint, J. (2008). Neuregulin 1 genotype and schizophrenia. *Schizophr Bull, 34*(1), 9-12. doi: 10.1093/schbul/sbm129

Ohi, K., Hashimoto, R., Yasuda, Y., Yoshida, T., Takahashi, H., Iike, N., . . . Takeda, M. (2010). The chitinase 3-like 1 gene and schizophrenia: evidence from a multi-center case-control study and meta-analysis. *Schizophr Res, 116*(2-3), 126-132. doi: 10.1016/j.schres.2009.12.002

Okochi, T., Ikeda, M., Kishi, T., Kawashima, K., Kinoshita, Y., Kitajima, T., . . . Iwata, N. (2009). Meta-analysis of association between genetic variants in COMT and schizophrenia: an update. *Schizophr Res, 110*(1-3), 140-148. doi: 10.1016/j.schres.2009.02.019

Shi b, J., Gershon, E. S., & Liu, C. (2008). Genetic associations with schizophrenia: meta-analyses of 12 candidate genes. *Schizophr Res, 104*(1-3), 96-107. doi: 10.1016/j.schres.2008.06.016

Shi, J., Badner, J. A., Gershon, E. S., & Liu, C. (2008). Allelic association of G72/G30 with schizophrenia and bipolar disorder: a comprehensive meta-analysis. *Schizophr Res, 98*(1-3), 89-97. doi: 10.1016/j.schres.2007.10.004

Zhang, R., Yan, J. D., Valenzuela, R. K., Lu, S. M., Du, X. Y., Zhong, B., . . . Ma, J. (2012). Further evidence for the association of genetic variants of ZNF804A with schizophrenia and a meta-analysis for genome-wide significance variant rs1344706. *Schizophr Res, 141*(1), 40-47. doi: 10.1016/j.schres.2012.07.013

**Alzheimer Disease**

**Primary studies**

Bullido, M. J., Artiga, M. J., Recuero, M., Sastre, I., Garcia, M. A., Aldudo, J., . . . Valdivieso, F. (1998). A polymorphism in the regulatory region of APOE associated with risk for Alzheimer's dementia. *Nat Genet, 18*(1), 69-71. doi: 10.1038/ng0198-69

Clarke, R., Smith, A. D., Jobst, K. A., Refsum, H., Sutton, L., & Ueland, P. M. (1998). Folate, vitamin B12, and serum total homocysteine levels in confirmed Alzheimer disease. *Arch Neurol, 55*(11), 1449-1455.

Forette, F., Seux, M. L., Staessen, J. A., Thijs, L., Birkenhager, W. H., Babarskiene, M. R., . . . Fagard, R. (1998). Prevention of dementia in randomised double-blind placebo-controlled Systolic Hypertension in Europe (Syst-Eur) trial. *Lancet, 352*(9137), 1347-1351.

Grupe, A., Abraham, R., Li, Y., Rowland, C., Hollingworth, P., Morgan, A., . . . Williams, J. (2007). Evidence for novel susceptibility genes for late-onset Alzheimer's disease from a genome-wide association study of putative functional variants. *Hum Mol Genet, 16*(8), 865-873. doi: 10.1093/hmg/ddm031

Harold, D., Abraham, R., Hollingworth, P., Sims, R., Gerrish, A., Hamshere, M. L., . . . Williams, J. (2009). Genome-wide association study identifies variants at CLU and PICALM associated with Alzheimer's disease. *Nat Genet, 41*(10), 1088-1093. doi: 10.1038/ng.440

Khachaturian, A. S., Zandi, P. P., Lyketsos, C. G., Hayden, K. M., Skoog, I., Norton, M. C., . . . Breitner, J. C. (2006). Antihypertensive medication use and incident Alzheimer disease: the Cache County Study. *Arch Neurol, 63*(5), 686-692. doi: 10.1001/archneur.63.5.noc60013

Lambert, J. C., Heath, S., Even, G., Campion, D., Sleegers, K., Hiltunen, M., . . . Amouyel, P. (2009). Genome-wide association study identifies variants at CLU and CR1 associated with Alzheimer's disease. *Nat Genet, 41*(10), 1094-1099. doi: 10.1038/ng.439

Papassotiropoulos, A., Streffer, J. R., Tsolaki, M., Schmid, S., Thal, D., Nicosia, F., . . . Hock, C. (2003). Increased brain beta-amyloid load, phosphorylated tau, and risk of Alzheimer disease associated with an intronic CYP46 polymorphism. *Arch Neurol, 60*(1), 29-35.

Peters, R., Beckett, N., Forette, F., Tuomilehto, J., Clarke, R., Ritchie, C., . . . Bulpitt, C. (2008). Incident dementia and blood pressure lowering in the Hypertension in the Very Elderly Trial cognitive function assessment (HYVET-COG): a double-blind, placebo controlled trial. *Lancet Neurol, 7*(8), 683-689. doi: 10.1016/s1474-4422(08)70143-1

Reiman, E. M., Webster, J. A., Myers, A. J., Hardy, J., Dunckley, T., Zismann, V. L., . . . Stephan, D. A. (2007). GAB2 alleles modify Alzheimer's risk in APOE epsilon4 carriers. *Neuron, 54*(5), 713-720. doi: 10.1016/j.neuron.2007.05.022

Rogaeva, E., Meng, Y., Lee, J. H., Gu, Y., Kawarai, T., Zou, F., . . . St George-Hyslop, P. (2007). The neuronal sortilin-related receptor SORL1 is genetically associated with Alzheimer disease. *Nat Genet, 39*(2), 168-177. doi: 10.1038/ng1943

Strittmatter, W. J., Saunders, A. M., Schmechel, D., Pericak-Vance, M., Enghild, J., Salvesen, G. S., & Roses, A. D. (1993). Apolipoprotein E: high-avidity binding to beta-amyloid and increased frequency of type 4 allele in late-onset familial Alzheimer disease. *Proc Natl Acad Sci U S A, 90*(5), 1977-1981.

**Meta-analysis**

Antunez, C., Boada, M., Lopez-Arrieta, J., Moreno-Rey, C., Hernandez, I., Marin, J., . . . Ruiz, A. (2011). Genetic association of complement receptor 1 polymorphism rs3818361 in Alzheimer's disease. *Alzheimers Dement, 7*(4), e124-129. doi: 10.1016/j.jalz.2011.05.2412

Belbin, O., Carrasquillo, M. M., Crump, M., Culley, O. J., Hunter, T. A., Ma, L., . . . Younkin, S. G. (2011). Investigation of 15 of the top candidate genes for late-onset Alzheimer's disease. *Hum Genet, 129*(3), 273-282. doi: 10.1007/s00439-010-0924-2

Chang-Quan, H., Hui, W., Chao-Min, W., Zheng-Rong, W., Jun-Wen, G., Yong-Hong, L., . . . Qing-Xiu, L. (2011). The association of antihypertensive medication use with risk of cognitive decline and dementia: a meta-analysis of longitudinal studies. *Int J Clin Pract, 65*(12), 1295-1305. doi: 10.1111/j.1742-1241.2011.02810.x

Ho, R. C., Cheung, M. W., Fu, E., Win, H. H., Zaw, M. H., Ng, A., & Mak, A. (2011). Is high homocysteine level a risk factor for cognitive decline in elderly? A systematic review, meta-analysis, and meta-regression. *Am J Geriatr Psychiatry, 19*(7), 607-617. doi: 10.1097/JGP.0b013e3181f17eed

Li b, L., Yin, Z., Liu, J., Li, G., Wang, Y., Yan, J., & Zhou, H. (2013). CYP46A1 T/C polymorphism associated with the APOE epsilon4 allele increases the risk of Alzheimer's disease. *J Neurol, 260*(7), 1701-1708. doi: 10.1007/s00415-012-6690-4

Reynolds, C. A., Hong, M. G., Eriksson, U. K., Blennow, K., Johansson, B., Malmberg, B., . . . Prince, J. A. (2010). Sequence variation in SORL1 and dementia risk in Swedes. *Neurogenetics, 11*(1), 139-142. doi: 10.1007/s10048-009-0210-4

Sadigh-Eteghad, S., Talebi, M., & Farhoudi, M. (2012). Association of apolipoprotein E epsilon 4 allele with sporadic late onset Alzheimer`s disease. A meta-analysis. *Neurosciences (Riyadh), 17*(4), 321-326.

Xin, X. Y., Ding, J. Q., & Chen, S. D. (2010). Apolipoprotein E promoter polymorphisms and risk of Alzheimer's disease: evidence from meta-analysis. *J Alzheimers Dis, 19*(4), 1283-1294. doi: 10.3233/jad-2010-1329

**Multiple Sclerosis**

**Primary studies**

Alotaibi, S., Kennedy, J., Tellier, R., Stephens, D., & Banwell, B. (2004). Epstein-Barr virus in pediatric multiple sclerosis. *Jama, 291*(15), 1875-1879. doi: 10.1001/jama.291.15.1875

ANZgene. (2009). Genome-wide association study identifies new multiple sclerosis susceptibility loci on chromosomes 12 and 20. *Nat Genet, 41*(7), 824-828. doi: 10.1038/ng.396

Ascherio, A., Munger, K. L., Lennette, E. T., Spiegelman, D., Hernan, M. A., Olek, M. J., . . . Hunter, D. J. (2001). Epstein-Barr virus antibodies and risk of multiple sclerosis: a prospective study. *Jama, 286*(24), 3083-3088.

Ascherio, A., Zhang, S. M., Hernan, M. A., Olek, M. J., Coplan, P. M., Brodovicz, K., & Walker, A. M. (2001). Hepatitis B vaccination and the risk of multiple sclerosis. *N Engl J Med, 344*(5), 327-332. doi: 10.1056/nejm200102013440502

Aulchenko, Y. S., Hoppenbrouwers, I. A., Ramagopalan, S. V., Broer, L., Jafari, N., Hillert, J., . . . Hintzen, R. Q. (2008). Genetic variation in the KIF1B locus influences susceptibility to multiple sclerosis. *Nat Genet, 40*(12), 1402-1403. doi: 10.1038/ng.251

Gregory, S. G., Schmidt, S., Seth, P., Oksenberg, J. R., Hart, J., Prokop, A., . . . Haines, J. L. (2007). Interleukin 7 receptor alpha chain (IL7R) shows allelic and functional association with multiple sclerosis. *Nat Genet, 39*(9), 1083-1091. doi: 10.1038/ng2103

Hafler, D. A., Compston, A., Sawcer, S., Lander, E. S., Daly, M. J., De Jager, P. L., . . . Hauser, S. L. (2007). Risk alleles for multiple sclerosis identified by a genomewide study. *N Engl J Med, 357*(9), 851-862. doi: 10.1056/NEJMoa073493

Hedstrom, A. K., Baarnhielm, M., Olsson, T., & Alfredsson, L. (2009). Tobacco smoking, but not Swedish snuff use, increases the risk of multiple sclerosis. *Neurology, 73*(9), 696-701. doi: 10.1212/WNL.0b013e3181b59c40

Hernan, M. A., Jick, S. S., Logroscino, G., Olek, M. J., Ascherio, A., & Jick, H. (2005). Cigarette smoking and the progression of multiple sclerosis. *Brain, 128*(Pt 6), 1461-1465. doi: 10.1093/brain/awh471

Hernan, M. A., Jick, S. S., Olek, M. J., & Jick, H. (2004). Recombinant hepatitis B vaccine and the risk of multiple sclerosis: a prospective study. *Neurology, 63*(5), 838-842.

Ponsonby, A. L., van der Mei, I., Dwyer, T., Blizzard, L., Taylor, B., Kemp, A., . . . Kilpatrick, T. (2005). Exposure to infant siblings during early life and risk of multiple sclerosis. *Jama, 293*(4), 463-469. doi: 10.1001/jama.293.4.463

Riise, T., Nortvedt, M. W., & Ascherio, A. (2003). Smoking is a risk factor for multiple sclerosis. *Neurology, 61*(8), 1122-1124.

Zhang, R., Duan, L., Jiang, Y., Zhang, X., Sun, P., Li, J., . . . Li, X. (2011). Association between the IL7R T244I polymorphism and multiple sclerosis: a meta-analysis. *Mol Biol Rep, 38*(8), 5079-5084. doi: 10.1007/s11033-010-0654-5

**Meta-analysis**

Farez, M. F., & Correale, J. (2011). Immunizations and risk of multiple sclerosis: systematic review and meta-analysis. *J Neurol, 258*(7), 1197-1206. doi: 10.1007/s00415-011-5984-2

Handel, A. E., Williamson, A. J., Disanto, G., Dobson, R., Giovannoni, G., & Ramagopalan, S. V. (2011). Smoking and multiple sclerosis: an updated meta-analysis. *PLoS One, 6*(1), e16149. doi: 10.1371/journal.pone.0016149

Handel, A. E., Williamson, A. J., Disanto, G., Handunnetthi, L., Giovannoni, G., & Ramagopalan, S. V. (2010). An updated meta-analysis of risk of multiple sclerosis following infectious mononucleosis. *PLoS One, 5*(9). doi: 10.1371/journal.pone.0012496

Kudryavtseva, E. A., Rozhdestvenskii, A. S., Kakulya, A. V., Khanokh, E. V., Delov, R. A., Malkova, N. A., . . . Filipenko, M. L. (2011). Polymorphic locus rs10492972 of the KIF1B gene association with multiple sclerosis in Russia: case control study. *Mol Genet Metab, 104*(3), 390-394. doi: 10.1016/j.ymgme.2011.05.018

Santiago, O., Gutierrez, J., Sorlozano, A., de Dios Luna, J., Villegas, E., & Fernandez, O. (2010). Relation between Epstein-Barr virus and multiple sclerosis: analytic study of scientific production. *Eur J Clin Microbiol Infect Dis, 29*(7), 857-866. doi: 10.1007/s10096-010-0940-0

**Parkinson Disease**

**Primary studies**

Ascherio, A., Chen, H., Weisskopf, M. G., O'Reilly, E., McCullough, M. L., Calle, E. E., . . . Thun, M. J. (2006). Pesticide exposure and risk for Parkinson's disease. *Ann Neurol, 60*(2), 197-203. doi: 10.1002/ana.20904

Bower, J. H., Maraganore, D. M., Peterson, B. J., McDonnell, S. K., Ahlskog, J. E., & Rocca, W. A. (2003). Head trauma preceding PD: a case-control study. *Neurology, 60*(10), 1610-1615.

Chen, H., Huang, X., Guo, X., Mailman, R. B., Park, Y., Kamel, F., . . . Blair, A. (2010). Smoking duration, intensity, and risk of Parkinson disease. *Neurology, 74*(11), 878-884. doi: 10.1212/WNL.0b013e3181d55f38

Chen, H., Zhang, S. M., Hernan, M. A., Schwarzschild, M. A., Willett, W. C., Colditz, G. A., . . . Ascherio, A. (2003). Nonsteroidal anti-inflammatory drugs and the risk of Parkinson disease. *Arch Neurol, 60*(8), 1059-1064. doi: 10.1001/archneur.60.8.1059

Clark, L. N., Ross, B. M., Wang, Y., Mejia-Santana, H., Harris, J., Louis, E. D., . . . Marder, K. (2007). Mutations in the glucocerebrosidase gene are associated with early-onset Parkinson disease. *Neurology, 69*(12), 1270-1277. doi: 10.1212/01.wnl.0000276989.17578.02

Gao, X., Simon, K. C., Schwarzschild, M. A., & Ascherio, A. (2012). Prospective study of statin use and risk of Parkinson disease. *Arch Neurol, 69*(3), 380-384. doi: 10.1001/archneurol.2011.1060

Gilks, W. P., Abou-Sleiman, P. M., Gandhi, S., Jain, S., Singleton, A., Lees, A. J., . . . Wood, N. W. (2005). A common LRRK2 mutation in idiopathic Parkinson's disease. *Lancet, 365*(9457), 415-416. doi: 10.1016/s0140-6736(05)17830-1

Koller, W. C., Glatt, S. L., Hubble, J. P., Paolo, A., Troster, A. I., Handler, M. S., . . . et al. (1995). Apolipoprotein E genotypes in Parkinson's disease with and without dementia. *Ann Neurol, 37*(2), 242-245. doi: 10.1002/ana.410370215

Lesage, S., Durr, A., Tazir, M., Lohmann, E., Leutenegger, A. L., Janin, S., . . . Brice, A. (2006). LRRK2 G2019S as a cause of Parkinson's disease in North African Arabs. *N Engl J Med, 354*(4), 422-423. doi: 10.1056/NEJMc055540

Ozelius, L. J., Senthil, G., Saunders-Pullman, R., Ohmann, E., Deligtisch, A., Tagliati, M., . . . Bressman, S. B. (2006). LRRK2 G2019S as a cause of Parkinson's disease in Ashkenazi Jews. *N Engl J Med, 354*(4), 424-425. doi: 10.1056/NEJMc055509

Ross, G. W., Abbott, R. D., Petrovitch, H., Morens, D. M., Grandinetti, A., Tung, K. H., . . . White, L. R. (2000). Association of coffee and caffeine intake with the risk of Parkinson disease. *Jama, 283*(20), 2674-2679.

Ross, O. A., Wu, Y. R., Lee, M. C., Funayama, M., Chen, M. L., Soto, A. I., . . . Wu, R. M. (2008). Analysis of Lrrk2 R1628P as a risk factor for Parkinson's disease. *Ann Neurol, 64*(1), 88-92. doi: 10.1002/ana.21405

Sidransky, E., Nalls, M. A., Aasly, J. O., Aharon-Peretz, J., Annesi, G., Barbosa, E. R., . . . Ziegler, S. G. (2009). Multicenter analysis of glucocerebrosidase mutations in Parkinson's disease. *N Engl J Med, 361*(17), 1651-1661. doi: 10.1056/NEJMoa0901281

Sobel, N., Thomason, M. E., Stappen, I., Tanner, C. M., Tetrud, J. W., Bower, J. M., . . . Gabrieli, J. D. (2001). An impairment in sniffing contributes to the olfactory impairment in Parkinson's disease. *Proc Natl Acad Sci U S A, 98*(7), 4154-4159. doi: 10.1073/pnas.071061598

Tanner, C. M., Ross, G. W., Jewell, S. A., Hauser, R. A., Jankovic, J., Factor, S. A., . . . Langston, J. W. (2009). Occupation and risk of parkinsonism: a multicenter case-control study. *Arch Neurol, 66*(9), 1106-1113. doi: 10.1001/archneurol.2009.195

Thacker, E. L., O'Reilly, E. J., Weisskopf, M. G., Chen, H., Schwarzschild, M. A., McCullough, M. L., . . . Ascherio, A. (2007). Temporal relationship between cigarette smoking and risk of Parkinson disease. *Neurology, 68*(10), 764-768. doi: 10.1212/01.wnl.0000256374.50227.4b

Wahner, A. D., Sinsheimer, J. S., Bronstein, J. M., & Ritz, B. (2007). Inflammatory cytokine gene polymorphisms and increased risk of Parkinson disease. *Arch Neurol, 64*(6), 836-840. doi: 10.1001/archneur.64.6.836

**Meta-analysis**

Lill, C. M., Roehr, J. T., McQueen, M. B., Kavvoura, F. K., Bagade, S., Schjeide, B. M., . . . Bertram, L. (2012). Comprehensive research synopsis and systematic meta-analyses in Parkinson's disease genetics: The PDGene database. *PLoS Genet, 8*(3), e1002548. doi: 10.1371/journal.pgen.1002548

Noyce, A. J., Bestwick, J. P., Silveira-Moriyama, L., Hawkes, C. H., Giovannoni, G., Lees, A. J., & Schrag, A. (2012). Meta-analysis of early nonmotor features and risk factors for Parkinson disease. *Ann Neurol, 72*(6), 893-901. doi: 10.1002/ana.23687

Rahayel, S., Frasnelli, J., & Joubert, S. (2012). The effect of Alzheimer's disease and Parkinson's disease on olfaction: a meta-analysis. *Behav Brain Res, 231*(1), 60-74. doi: 10.1016/j.bbr.2012.02.047

Undela, K., Gudala, K., Malla, S., & Bansal, D. (2013). Statin use and risk of Parkinson's disease: a meta-analysis of observational studies. *J Neurol, 260*(1), 158-165. doi: 10.1007/s00415-012-6606-3

van der Mark, M., Brouwer, M., Kromhout, H., Nijssen, P., Huss, A., & Vermeulen, R. (2012). Is pesticide use related to Parkinson disease? Some clues to heterogeneity in study results. *Environ Health Perspect, 120*(3), 340-347. doi: 10.1289/ehp.1103881

Wu, X., Tang, K. F., Li, Y., Xiong, Y. Y., Shen, L., Wei, Z. Y., . . . Qin, S. Y. (2012). Quantitative assessment of the effect of LRRK2 exonic variants on the risk of Parkinson's disease: a meta-analysis. *Parkinsonism Relat Disord, 18*(6), 722-730. doi: 10.1016/j.parkreldis.2012.04.013

**Breast Cancer**

**Primary studies**

Ahlgren, M., Melbye, M., Wohlfahrt, J., & Sorensen, T. I. (2004). Growth patterns and the risk of breast cancer in women. *N Engl J Med, 351*(16), 1619-1626. doi: 10.1056/NEJMoa040576

Ahmed, S., Thomas, G., Ghoussaini, M., Healey, C. S., Humphreys, M. K., Platte, R., . . . Easton, D. F. (2009). Newly discovered breast cancer susceptibility loci on 3p24 and 17q23.2. *Nat Genet, 41*(5), 585-590. doi: 10.1038/ng.354

Ambrosone, C. B., Freudenheim, J. L., Graham, S., Marshall, J. R., Vena, J. E., Brasure, J. R., . . . Shields, P. G. (1996). Cigarette smoking, N-acetyltransferase 2 genetic polymorphisms, and breast cancer risk. *Jama, 276*(18), 1494-1501.

Bardia, A., Hartmann, L. C., Vachon, C. M., Vierkant, R. A., Wang, A. H., Olson, J. E., . . . Cerhan, J. R. (2006). Recreational physical activity and risk of postmenopausal breast cancer based on hormone receptor status. *Arch Intern Med, 166*(22), 2478-2483. doi: 10.1001/archinte.166.22.2478

Bonnet, M., Guinebretiere, J. M., Kremmer, E., Grunewald, V., Benhamou, E., Contesso, G., & Joab, I. (1999). Detection of Epstein-Barr virus in invasive breast cancers. *J Natl Cancer Inst, 91*(16), 1376-1381.

Boudreau, D. M., Gardner, J. S., Malone, K. E., Heckbert, S. R., Blough, D. K., & Daling, J. R. (2004). The association between 3-hydroxy-3-methylglutaryl conenzyme A inhibitor use and breast carcinoma risk among postmenopausal women: a case-control study. *Cancer, 100*(11), 2308-2316. doi: 10.1002/cncr.20271

Butler, L. M., Wu, A. H., Wang, R., Koh, W. P., Yuan, J. M., & Yu, M. C. (2010). A vegetable-fruit-soy dietary pattern protects against breast cancer among postmenopausal Singapore Chinese women. *Am J Clin Nutr, 91*(4), 1013-1019. doi: 10.3945/ajcn.2009.28572

Cade, J. E., Burley, V. J., & Greenwood, D. C. (2007). Dietary fibre and risk of breast cancer in the UK Women's Cohort Study. *Int J Epidemiol, 36*(2), 431-438. doi: 10.1093/ije/dyl295

Cauley, J. A., McTiernan, A., Rodabough, R. J., LaCroix, A., Bauer, D. C., Margolis, K. L., . . . Chlebowski, R. T. (2006). Statin use and breast cancer: prospective results from the Women's Health Initiative. *J Natl Cancer Inst, 98*(10), 700-707. doi: 10.1093/jnci/djj188

CHEK2-BCC. (2004). CHEK2*1100delC and susceptibility to breast cancer: a collaborative analysis involving 10,860 breast cancer cases and 9,065 controls from 10 studies. *Am J Hum Genet, 74*(6), 1175-1182. doi: 10.1086/421251

Cho, E., Chen, W. Y., Hunter, D. J., Stampfer, M. J., Colditz, G. A., Hankinson, S. E., & Willett, W. C. (2006). Red meat intake and risk of breast cancer among premenopausal women. *Arch Intern Med, 166*(20), 2253-2259. doi: 10.1001/archinte.166.20.2253

Cohn, B. A., Cirillo, P. M., Christianson, R. E., van den Berg, B. J., & Siiteri, P. K. (2001). Placental characteristics and reduced risk of maternal breast cancer. *J Natl Cancer Inst, 93*(15), 1133-1140.

Cook, N. R., Lee, I. M., Gaziano, J. M., Gordon, D., Ridker, P. M., Manson, J. E., . . . Buring, J. E. (2005). Low-dose aspirin in the primary prevention of cancer: the Women's Health Study: a randomized controlled trial. *Jama, 294*(1), 47-55. doi: 10.1001/jama.294.1.47

Cristofanilli, M., Yamamura, Y., Kau, S. W., Bevers, T., Strom, S., Patangan, M., . . . Hortobagyi, G. N. (2005). Thyroid hormone and breast carcinoma. Primary hypothyroidism is associated with a reduced incidence of primary breast carcinoma. *Cancer, 103*(6), 1122-1128. doi: 10.1002/cncr.20881

Cybulski, C., Wokolorczyk, D., Jakubowska, A., Huzarski, T., Byrski, T., Gronwald, J., . . . Lubinski, J. (2011). Risk of breast cancer in women with a CHEK2 mutation with and without a family history of breast cancer. *J Clin Oncol, 29*(28), 3747-3752. doi: 10.1200/jco.2010.34.0778

Dallal, C. M., Sullivan-Halley, J., Ross, R. K., Wang, Y., Deapen, D., Horn-Ross, P. L., . . . Bernstein, L. (2007). Long-term recreational physical activity and risk of invasive and in situ breast cancer: the California teachers study. *Arch Intern Med, 167*(4), 408-415. doi: 10.1001/archinte.167.4.408

Dumeaux, V., Alsaker, E., & Lund, E. (2003). Breast cancer and specific types of oral contraceptives: a large Norwegian cohort study. *Int J Cancer, 105*(6), 844-850. doi: 10.1002/ijc.11167

Easton, D. F., Pooley, K. A., Dunning, A. M., Pharoah, P. D., Thompson, D., Ballinger, D. G., . . . Ponder, B. A. (2007). Genome-wide association study identifies novel breast cancer susceptibility loci. *Nature, 447*(7148), 1087-1093. doi: 10.1038/nature05887

Egan, K. M., Stampfer, M. J., Giovannucci, E., Rosner, B. A., & Colditz, G. A. (1996). Prospective study of regular aspirin use and the risk of breast cancer. *J Natl Cancer Inst, 88*(14), 988-993.

Eliassen, A. H., Colditz, G. A., Rosner, B., Willett, W. C., & Hankinson, S. E. (2005). Serum lipids, lipid-lowering drugs, and the risk of breast cancer. *Arch Intern Med, 165*(19), 2264-2271. doi: 10.1001/archinte.165.19.2264

Feigelson, H. S., Coetzee, G. A., Kolonel, L. N., Ross, R. K., & Henderson, B. E. (1997). A polymorphism in the CYP17 gene increases the risk of breast cancer. *Cancer Res, 57*(6), 1063-1065.

Fletcher, O., Johnson, N., Orr, N., Hosking, F. J., Gibson, L. J., Walker, K., . . . Peto, J. (2011). Novel breast cancer susceptibility locus at 9q31.2: results of a genome-wide association study. *J Natl Cancer Inst, 103*(5), 425-435. doi: 10.1093/jnci/djq563

Garcia Rodriguez, L. A., & Gonzalez-Perez, A. (2004). Risk of breast cancer among users of aspirin and other anti-inflammatory drugs. *Br J Cancer, 91*(3), 525-529. doi: 10.1038/sj.bjc.6602003

Gierach, G. L., Lacey, J. V., Jr., Schatzkin, A., Leitzmann, M. F., Richesson, D., Hollenbeck, A. R., & Brinton, L. A. (2008). Nonsteroidal anti-inflammatory drugs and breast cancer risk in the National Institutes of Health-AARP Diet and Health Study. *Breast Cancer Res, 10*(2), R38. doi: 10.1186/bcr2089

Gridley, G., McLaughlin, J. K., Ekbom, A., Klareskog, L., Adami, H. O., Hacker, D. G., . . . Fraumeni, J. F., Jr. (1993). Incidence of cancer among patients with rheumatoid arthritis. *J Natl Cancer Inst, 85*(4), 307-311.

Hannaford, P. C., Selvaraj, S., Elliott, A. M., Angus, V., Iversen, L., & Lee, A. J. (2007). Cancer risk among users of oral contraceptives: cohort data from the Royal College of General Practitioner's oral contraception study. *Bmj, 335*(7621), 651. doi: 10.1136/bmj.39289.649410.55

Harris, R. E., Namboodiri, K. K., & Farrar, W. B. (1996). Nonsteroidal antiinflammatory drugs and breast cancer. *Epidemiology, 7*(2), 203-205.

Hjartaker, A., Laake, P., & Lund, E. (2001). Childhood and adult milk consumption and risk of premenopausal breast cancer in a cohort of 48,844 women - the Norwegian women and cancer study. *Int J Cancer, 93*(6), 888-893.

Huang, Z., Hankinson, S. E., Colditz, G. A., Stampfer, M. J., Hunter, D. J., Manson, J. E., . . . Willett, W. C. (1997). Dual effects of weight and weight gain on breast cancer risk. *Jama, 278*(17), 1407-1411.

Hunter, D. J., Kraft, P., Jacobs, K. B., Cox, D. G., Yeager, M., Hankinson, S. E., . . . Chanock, S. J. (2007). A genome-wide association study identifies alleles in FGFR2 associated with risk of sporadic postmenopausal breast cancer. *Nat Genet, 39*(7), 870-874. doi: 10.1038/ng2075

Hunter, D. J., Manson, J. E., Colditz, G. A., Stampfer, M. J., Rosner, B., Hennekens, C. H., . . . Willett, W. C. (1993). A prospective study of the intake of vitamins C, E, and A and the risk of breast cancer. *N Engl J Med, 329*(4), 234-240. doi: 10.1056/nejm199307223290403

Ingram, D., Sanders, K., Kolybaba, M., & Lopez, D. (1997). Case-control study of phyto-oestrogens and breast cancer. *Lancet, 350*(9083), 990-994. doi: 10.1016/s0140-6736(97)01339-1

Jacobs, E. J., Thun, M. J., Bain, E. B., Rodriguez, C., Henley, S. J., & Calle, E. E. (2007). A large cohort study of long-term daily use of adult-strength aspirin and cancer incidence. *J Natl Cancer Inst, 99*(8), 608-615. doi: 10.1093/jnci/djk132

Knekt, P., Jarvinen, R., Seppanen, R., Pukkala, E., & Aromaa, A. (1996). Intake of dairy products and the risk of breast cancer. *Br J Cancer, 73*(5), 687-691.

Krontiris, T. G., Devlin, B., Karp, D. D., Robert, N. J., & Risch, N. (1993). An association between the risk of cancer and mutations in the HRAS1 minisatellite locus. *N Engl J Med, 329*(8), 517-523. doi: 10.1056/nejm199308193290801

Lee, H. P., Gourley, L., Duffy, S. W., Esteve, J., Lee, J., & Day, N. E. (1991). Dietary effects on breast-cancer risk in Singapore. *Lancet, 337*(8751), 1197-1200.

Leitzmann, M. F., Moore, S. C., Peters, T. M., Lacey, J. V., Jr., Schatzkin, A., Schairer, C., . . . Albanes, D. (2008). Prospective study of physical activity and risk of postmenopausal breast cancer. *Breast Cancer Res, 10*(5), R92. doi: 10.1186/bcr2190

London, S. J., Pogoda, J. M., Hwang, K. L., Langholz, B., Monroe, K. R., Kolonel, L. N., . . . Henderson, B. E. (2003). Residential magnetic field exposure and breast cancer risk: a nested case-control study from a multiethnic cohort in Los Angeles County, California. *Am J Epidemiol, 158*(10), 969-980.

Marshall, S. F., Bernstein, L., Anton-Culver, H., Deapen, D., Horn-Ross, P. L., Mohrenweiser, H., . . . Ross, R. K. (2005). Nonsteroidal anti-inflammatory drug use and breast cancer risk by stage and hormone receptor status. *J Natl Cancer Inst, 97*(11), 805-812. doi: 10.1093/jnci/dji140

Maruti, S. S., Willett, W. C., Feskanich, D., Rosner, B., & Colditz, G. A. (2008). A prospective study of age-specific physical activity and premenopausal breast cancer. *J Natl Cancer Inst, 100*(10), 728-737. doi: 10.1093/jnci/djn135

McTiernan, A., Kooperberg, C., White, E., Wilcox, S., Coates, R., Adams-Campbell, L. L., . . . Ockene, J. (2003). Recreational physical activity and the risk of breast cancer in postmenopausal women: the Women's Health Initiative Cohort Study. *Jama, 290*(10), 1331-1336. doi: 10.1001/jama.290.10.1331

Meijers-Heijboer, H., van den Ouweland, A., Klijn, J., Wasielewski, M., de Snoo, A., Oldenburg, R., . . . Stratton, M. R. (2002). Low-penetrance susceptibility to breast cancer due to CHEK2(*)1100delC in noncarriers of BRCA1 or BRCA2 mutations. *Nat Genet, 31*(1), 55-59. doi: 10.1038/ng879

Michels, K. B., Terry, K. L., & Willett, W. C. (2006). Longitudinal study on the role of body size in premenopausal breast cancer. *Arch Intern Med, 166*(21), 2395-2402. doi: 10.1001/archinte.166.21.2395

Paltiel, O., Friedlander, Y., Tiram, E., Barchana, M., Xue, X., & Harlap, S. (2004). Cancer after pre-eclampsia: follow up of the Jerusalem perinatal study cohort. *Bmj, 328*(7445), 919. doi: 10.1136/bmj.38032.820451.7C

Park, Y., Brinton, L. A., Subar, A. F., Hollenbeck, A., & Schatzkin, A. (2009). Dietary fiber intake and risk of breast cancer in postmenopausal women: the National Institutes of Health-AARP Diet and Health Study. *Am J Clin Nutr, 90*(3), 664-671. doi: 10.3945/ajcn.2009.27758

Reeves, G. K., Pirie, K., Beral, V., Green, J., Spencer, E., & Bull, D. (2007). Cancer incidence and mortality in relation to body mass index in the Million Women Study: cohort study. *Bmj, 335*(7630), 1134. doi: 10.1136/bmj.39367.495995.AE

Rockhill, B., Willett, W. C., Hunter, D. J., Manson, J. E., Hankinson, S. E., & Colditz, G. A. (1999). A prospective study of recreational physical activity and breast cancer risk. *Arch Intern Med, 159*(19), 2290-2296.

Rockhill, B., Willett, W. C., Hunter, D. J., Manson, J. E., Hankinson, S. E., Spiegelman, D., & Colditz, G. A. (1998). Physical activity and breast cancer risk in a cohort of young women. *J Natl Cancer Inst, 90*(15), 1155-1160.

Schoenfeld, E. R., O'Leary, E. S., Henderson, K., Grimson, R., Kabat, G. C., Ahnn, S., . . . Leske, M. C. (2003). Electromagnetic fields and breast cancer on Long Island: a case-control study. *Am J Epidemiol, 158*(1), 47-58.

Smitten, A. L., Simon, T. A., Hochberg, M. C., & Suissa, S. (2008). A meta-analysis of the incidence of malignancy in adult patients with rheumatoid arthritis. *Arthritis Res Ther, 10*(2), R45. doi: 10.1186/ar2404

Stacey, S. N., Manolescu, A., Sulem, P., Thorlacius, S., Gudjonsson, S. A., Jonsson, G. F., . . . Stefansson, K. (2008). Common variants on chromosome 5p12 confer susceptibility to estrogen receptor-positive breast cancer. *Nat Genet, 40*(6), 703-706. doi: 10.1038/ng.131

Stattin, P., Bjor, O., Ferrari, P., Lukanova, A., Lenner, P., Lindahl, B., . . . Kaaks, R. (2007). Prospective study of hyperglycemia and cancer risk. *Diabetes Care, 30*(3), 561-567. doi: 10.2337/dc06-0922

Steindorf, K., Ritte, R., Eomois, P. P., Lukanova, A., Tjonneland, A., Johnsen, N. F., . . . Kaaks, R. (2013). Physical activity and risk of breast cancer overall and by hormone receptor status: the European prospective investigation into cancer and nutrition. *Int J Cancer, 132*(7), 1667-1678. doi: 10.1002/ijc.27778

Steingart, A., Cotterchio, M., Kreiger, N., & Sloan, M. (2003). Antidepressant medication use and breast cancer risk: a case-control study. *Int J Epidemiol, 32*(6), 961-966.

Suzuki, R., Ye, W., Rylander-Rudqvist, T., Saji, S., Colditz, G. A., & Wolk, A. (2005). Alcohol and postmenopausal breast cancer risk defined by estrogen and progesterone receptor status: a prospective cohort study. *J Natl Cancer Inst, 97*(21), 1601-1608. doi: 10.1093/jnci/dji341

Taylor, E. F., Burley, V. J., Greenwood, D. C., & Cade, J. E. (2007). Meat consumption and risk of breast cancer in the UK Women's Cohort Study. *Br J Cancer, 96*(7), 1139-1146. doi: 10.1038/sj.bjc.6603689

Terry, M. B., Gammon, M. D., Zhang, F. F., Tawfik, H., Teitelbaum, S. L., Britton, J. A., . . . Neugut, A. I. (2004). Association of frequency and duration of aspirin use and hormone receptor status with breast cancer risk. *Jama, 291*(20), 2433-2440. doi: 10.1001/jama.291.20.2433

Thompson, W. D., Jacobson, H. I., Negrini, B., & Janerich, D. T. (1989). Hypertension, pregnancy, and risk of breast cancer. *J Natl Cancer Inst, 81*(20), 1571-1574.

Thune, I., Brenn, T., Lund, E., & Gaard, M. (1997). Physical activity and the risk of breast cancer. *N Engl J Med, 336*(18), 1269-1275. doi: 10.1056/nejm199705013361801

Touillaud, M. S., Thiebaut, A. C., Fournier, A., Niravong, M., Boutron-Ruault, M. C., & Clavel-Chapelon, F. (2007). Dietary lignan intake and postmenopausal breast cancer risk by estrogen and progesterone receptor status. *J Natl Cancer Inst, 99*(6), 475-486. doi: 10.1093/jnci/djk096

Travis, R. C., Reeves, G. K., Green, J., Bull, D., Tipper, S. J., Baker, K., . . . Lathrop, M. (2010). Gene-environment interactions in 7610 women with breast cancer: prospective evidence from the Million Women Study. *Lancet, 375*(9732), 2143-2151. doi: 10.1016/s0140-6736(10)60636-8

Turnbull, C., Ahmed, S., Morrison, J., Pernet, D., Renwick, A., Maranian, M., . . . Easton, D. F. (2010). Genome-wide association study identifies five new breast cancer susceptibility loci. *Nat Genet, 42*(6), 504-507. doi: 10.1038/ng.586

van Gils, C. H., Peeters, P. H., Bueno-de-Mesquita, H. B., Boshuizen, H. C., Lahmann, P. H., Clavel-Chapelon, F., . . . Riboli, E. (2005). Consumption of vegetables and fruits and risk of breast cancer. *Jama, 293*(2), 183-193. doi: 10.1001/jama.293.2.183

Yamamoto, S., Sobue, T., Kobayashi, M., Sasaki, S., & Tsugane, S. (2003). Soy, isoflavones, and breast cancer risk in Japan. *J Natl Cancer Inst, 95*(12), 906-913.

Ziv, E., Cauley, J., Morin, P. A., Saiz, R., & Browner, W. S. (2001). Association between the T29-->C polymorphism in the transforming growth factor beta1 gene and breast cancer among elderly white women: The Study of Osteoporotic Fractures. *Jama, 285*(22), 2859-2863.

**Meta-analysis**

Ambrosone, C. B., Kropp, S., Yang, J., Yao, S., Shields, P. G., & Chang-Claude, J. (2008). Cigarette smoking, N-acetyltransferase 2 genotypes, and breast cancer risk: pooled analysis and meta-analysis. *Cancer Epidemiol Biomarkers Prev, 17*(1), 15-26. doi: 10.1158/1055-9965.epi-07-0598

Angelousi, A. G., Anagnostou, V. K., Stamatakos, M. K., Georgiopoulos, G. A., & Kontzoglou, K. C. (2012). Mechanisms in endocrinology: primary HT and risk for breast cancer: a systematic review and meta-analysis. *Eur J Endocrinol, 166*(3), 373-381. doi: 10.1530/eje-11-0838

Aune c, D., Chan, D. S., Vieira, A. R., Rosenblatt, D. A., Vieira, R., Greenwood, D. C., & Norat, T. (2012). Fruits, vegetables and breast cancer risk: a systematic review and meta-analysis of prospective studies. *Breast Cancer Res Treat, 134*(2), 479-493. doi: 10.1007/s10549-012-2118-1

Aune, D., Chan, D. S., Greenwood, D. C., Vieira, A. R., Rosenblatt, D. A., Vieira, R., & Norat, T. (2012). Dietary fiber and breast cancer risk: a systematic review and meta-analysis of prospective studies. *Ann Oncol, 23*(6), 1394-1402. doi: 10.1093/annonc/mdr589

Bosetti, C., Rosato, V., Gallus, S., Cuzick, J., & La Vecchia, C. (2012). Aspirin and cancer risk: a quantitative review to 2011. *Ann Oncol, 23*(6), 1403-1415. doi: 10.1093/annonc/mds113

Boyle, P., Koechlin, A., Pizot, C., Boniol, M., Robertson, C., Mullie, P., . . . Autier, P. (2013). Blood glucose concentrations and breast cancer risk in women without diabetes: a meta-analysis. *Eur J Nutr, 52*(5), 1533-1540. doi: 10.1007/s00394-012-0460-z

Buck, K., Zaineddin, A. K., Vrieling, A., Linseisen, J., & Chang-Claude, J. (2010). Meta-analyses of lignans and enterolignans in relation to breast cancer risk. *Am J Clin Nutr, 92*(1), 141-153. doi: 10.3945/ajcn.2009.28573

Chen b, W., Zhong, R., Ming, J., Zou, L., Zhu, B., Lu, X., . . . Huang, T. (2012). The SLC4A7 variant rs4973768 is associated with breast cancer risk: evidence from a case-control study and a meta-analysis. *Breast Cancer Res Treat, 136*(3), 847-857. doi: 10.1007/s10549-012-2309-9

Chen, C., Ma, X., Zhong, M., & Yu, Z. (2010). Extremely low-frequency electromagnetic fields exposure and female breast cancer risk: a meta-analysis based on 24,338 cases and 60,628 controls. *Breast Cancer Res Treat, 123*(2), 569-576. doi: 10.1007/s10549-010-0782-6

Chen d, Y., & Pei, J. (2010). Factors influencing the association between CYP17 T34C polymorphism and the risk of breast cancer: meta-regression and subgroup analysis. *Breast Cancer Res Treat, 122*(2), 471-481. doi: 10.1007/s10549-009-0690-9

Dong c, J. Y., & Qin, L. Q. (2011). Soy isoflavones consumption and risk of breast cancer incidence or recurrence: a meta-analysis of prospective studies. *Breast Cancer Res Treat, 125*(2), 315-323. doi: 10.1007/s10549-010-1270-8

Dong d, J. Y., Zhang, L., He, K., & Qin, L. Q. (2011). Dairy consumption and risk of breast cancer: a meta-analysis of prospective cohort studies. *Breast Cancer Res Treat, 127*(1), 23-31. doi: 10.1007/s10549-011-1467-5

Eom, C. S., Park, S. M., & Cho, K. H. (2012). Use of antidepressants and the risk of breast cancer: a meta-analysis. *Breast Cancer Res Treat, 136*(3), 635-645. doi: 10.1007/s10549-012-2307-y

Fulan, H., Changxing, J., Baina, W. Y., Wencui, Z., Chunqing, L., Fan, W., . . . Yashuang, Z. (2011). Retinol, vitamins A, C, and E and breast cancer risk: a meta-analysis and meta-regression. *Cancer Causes Control, 22*(10), 1383-1396. doi: 10.1007/s10552-011-9811-y

Huang, Y., Li, B., Qian, J., Xie, J., & Yu, L. (2010). TGF-beta1 29T/C polymorphism and breast cancer risk: a meta-analysis involving 25,996 subjects. *Breast Cancer Res Treat, 123*(3), 863-868. doi: 10.1007/s10549-010-0796-0

Huo, Q., Zhang, N., & Yang, Q. (2012). Epstein-Barr virus infection and sporadic breast cancer risk: a meta-analysis. *PLoS One, 7*(2), e31656. doi: 10.1371/journal.pone.0031656

Kim, J. S., Kang, E. J., Woo, O. H., Park, K. H., Woo, S. U., Yang, D. S., . . . Seo, J. H. (2013). The relationship between preeclampsia, pregnancy-induced hypertension and maternal risk of breast cancer: a meta-analysis. *Acta Oncol, 52*(8), 1643-1648. doi: 10.3109/0284186x.2012.750033

Liu, C., Wang, Y., Wang, Q. S., & Wang, Y. J. (2012). The CHEK2 I157T variant and breast cancer susceptibility: a systematic review and meta-analysis. *Asian Pac J Cancer Prev, 13*(4), 1355-1360.

Renehan, A. G., Tyson, M., Egger, M., Heller, R. F., & Zwahlen, M. (2008). Body-mass index and incidence of cancer: a systematic review and meta-analysis of prospective observational studies. *Lancet, 371*(9612), 569-578. doi: 10.1016/s0140-6736(08)60269-x

Suzuki, R., Orsini, N., Mignone, L., Saji, S., & Wolk, A. (2008). Alcohol intake and risk of breast cancer defined by estrogen and progesterone receptor status--a meta-analysis of epidemiological studies. *Int J Cancer, 122*(8), 1832-1841. doi: 10.1002/ijc.23184

Takkouche, B., Regueira-Mendez, C., & Etminan, M. (2008). Breast cancer and use of nonsteroidal anti-inflammatory drugs: a meta-analysis. *J Natl Cancer Inst, 100*(20), 1439-1447. doi: 10.1093/jnci/djn324

Taylor, V. H., Misra, M., & Mukherjee, S. D. (2009). Is red meat intake a risk factor for breast cancer among premenopausal women? *Breast Cancer Res Treat, 117*(1), 1-8. doi: 10.1007/s10549-009-0441-y

Undela, K., Srikanth, V., & Bansal, D. (2012). Statin use and risk of breast cancer: a meta-analysis of observational studies. *Breast Cancer Res Treat, 135*(1), 261-269. doi: 10.1007/s10549-012-2154

Wang, H., Yang, Z., & Zhang, H. (2013). Assessing interactions between the associations of fibroblast growth factor receptor 2 common genetic variants and hormone receptor status with breast cancer risk. *Breast Cancer Res Treat, 137*(2), 511-522. doi: 10.1007/s10549-012-2343-7

Weischer, M., Bojesen, S. E., Ellervik, C., Tybjaerg-Hansen, A., & Nordestgaard, B. G. (2008). CHEK2*1100delC genotyping for clinical assessment of breast cancer risk: meta-analyses of 26,000 patient cases and 27,000 controls. *J Clin Oncol, 26*(4), 542-548. doi: 10.1200/jco.2007.12.5922

Wu, Y., Zhang, D., & Kang, S. (2013). Physical activity and risk of breast cancer: a meta-analysis of prospective studies. *Breast Cancer Res Treat, 137*(3), 869-882. doi: 10.1007/s10549-012-2396-7

Xu, X., Dailey, A. B., Peoples-Sheps, M., Talbott, E. O., Li, N., & Roth, J. (2009). Birth weight as a risk factor for breast cancer: a meta-analysis of 18 epidemiological studies. *J Womens Health (Larchmt), 18*(8), 1169-1178. doi: 10.1089/jwh.2008.1034

Zhang b, C., Lv, G. Q., Yu, X. M., Gu, Y. L., Li, J. P., Du, L. F., & Zhou, P. (2011). Current evidence on the relationship between HRAS1 polymorphism and breast cancer risk: a meta-analysis. *Breast Cancer Res Treat, 128*(2), 467-472. doi: 10.1007/s10549-011-1344-2

Zhu, H., Lei, X., Feng, J., & Wang, Y. (2012). Oral contraceptive use and risk of breast cancer: a meta-analysis of prospective cohort studies. *Eur J Contracept Reprod Health Care, 17*(6), 402-414. doi: 10.3109/13625187.2012.715357

**Glaucoma**

**Primary studies**

Rezaie, T., Child, A., Hitchings, R., Brice, G., Miller, L., Coca-Prados, M., . . . Sarfarazi, M. (2002). Adult-onset primary open-angle glaucoma caused by mutations in optineurin. *Science, 295*(5557), 1077-1079. doi: 10.1126/science.1066901

Thorleifsson, G., Magnusson, K. P., Sulem, P., Walters, G. B., Gudbjartsson, D. F., Stefansson, H., . . . Stefansson, K. (2007). Common sequence variants in the LOXL1 gene confer susceptibility to exfoliation glaucoma. *Science, 317*(5843), 1397-1400. doi: 10.1126/science.1146554

**Meta-analysis**

Chen, H., Chen, L. J., Zhang, M., Gong, W., Tam, P. O., Lam, D. S., & Pang, C. P. (2010). Ethnicity-based subgroup meta-analysis of the association of LOXL1 polymorphisms with glaucoma. *Mol Vis, 16*, 167-177.

Cheng, J. W., Li, P., & Wei, R. L. (2010). Meta-analysis of association between optineurin gene and primary open-angle glaucoma. *Med Sci Monit, 16*(8), Cr369-377.

**Rheumatoid Arthritis**

**Primary studies**

Begovich, A. B., Carlton, V. E., Honigberg, L. A., Schrodi, S. J., Chokkalingam, A. P., Alexander, H. C., . . . Gregersen, P. K. (2004). A missense single-nucleotide polymorphism in a gene encoding a protein tyrosine phosphatase (PTPN22) is associated with rheumatoid arthritis. *Am J Hum Genet, 75*(2), 330-337. doi: 10.1086/422827

Hutchinson, D., Shepstone, L., Moots, R., Lear, J. T., & Lynch, M. P. (2001). Heavy cigarette smoking is strongly associated with rheumatoid arthritis (RA), particularly in patients without a family history of RA. *Ann Rheum Dis, 60*(3), 223-227.

Kochi, Y., Yamada, R., Suzuki, A., Harley, J. B., Shirasawa, S., Sawada, T., . . . Yamamoto, K. (2005). A functional variant in FCRL3, encoding Fc receptor-like 3, is associated with rheumatoid arthritis and several autoimmunities. *Nat Genet, 37*(5), 478-485. doi: 10.1038/ng1540

Lee, H. S., Korman, B. D., Le, J. M., Kastner, D. L., Remmers, E. F., Gregersen, P. K., & Bae, S. C. (2009). Genetic risk factors for rheumatoid arthritis differ in Caucasian and Korean populations. *Arthritis Rheum, 60*(2), 364-371. doi: 10.1002/art.24245

Reckner Olsson, A., Skogh, T., & Wingren, G. (2001). Comorbidity and lifestyle, reproductive factors, and environmental exposures associated with rheumatoid arthritis. *Ann Rheum Dis, 60*(10), 934-939.

Simkins, H. M., Merriman, M. E., Highton, J., Chapman, P. T., O'Donnell, J. L., Jones, P. B., . . . Merriman, T. R. (2005). Association of the PTPN22 locus with rheumatoid arthritis in a New Zealand Caucasian cohort. *Arthritis Rheum, 52*(7), 2222-2225. doi: 10.1002/art.21126

Stolt, P., Bengtsson, C., Nordmark, B., Lindblad, S., Lundberg, I., Klareskog, L., & Alfredsson, L. (2003). Quantification of the influence of cigarette smoking on rheumatoid arthritis: results from a population based case-control study, using incident cases. *Ann Rheum Dis, 62*(9), 835-841.

Swanberg, M., Lidman, O., Padyukov, L., Eriksson, P., Akesson, E., Jagodic, M., . . . Olsson, T. (2005). MHC2TA is associated with differential MHC molecule expression and susceptibility to rheumatoid arthritis, multiple sclerosis and myocardial infarction. *Nat Genet, 37*(5), 486-494. doi: 10.1038/ng1544

The WTCCC-Burton, P. R. (2007). Genome-wide association study of 14,000 cases of seven common diseases and 3,000 shared controls. *Nature, 447*(7145), 661-678. doi: 10.1038/nature05911

Thomson, W., Barton, A., Ke, X., Eyre, S., Hinks, A., Bowes, J., . . . Worthington, J. (2007). Rheumatoid arthritis association at 6q23. *Nat Genet, 39*(12), 1431-1433. doi: 10.1038/ng.2007.32

Tokuhiro, S., Yamada, R., Chang, X., Suzuki, A., Kochi, Y., Sawada, T., . . . Yamamoto, K. (2003). An intronic SNP in a RUNX1 binding site of SLC22A4, encoding an organic cation transporter, is associated with rheumatoid arthritis. *Nat Genet, 35*(4), 341-348. doi: 10.1038/ng1267

**Meta-analysis**

Bronson, P. G., Criswell, L. A., & Barcellos, L. F. (2008). The MHC2TA -168A/G polymorphism and risk for rheumatoid arthritis: a meta-analysis of 6861 patients and 9270 controls reveals no evidence for association. *Ann Rheum Dis, 67*(7), 933-936. doi: 10.1136/ard.2007.077099

Lee d, Y. H., Woo, J. H., Choi, S. J., Ji, J. D., & Song, G. G. (2010). Fc receptor-like 3 -169 C/T polymorphism and RA susceptibility: a meta-analysis. *Rheumatol Int, 30*(7), 947-953. doi: 10.1007/s00296-009-1082-5

Lee e, Y. H., Bae, S. C., Choi, S. J., Ji, J. D., & Song, G. G. (2012). Associations between TNFAIP3 gene polymorphisms and rheumatoid arthritis: a meta-analysis. *Inflamm Res, 61*(6), 635-641. doi: 10.1007/s00011-012-0455-5

Lee g, Y. H., Bae, S. C., Choi, S. J., Ji, J. D., & Song, G. G. (2012). The association between the PTPN22 C1858T polymorphism and rheumatoid arthritis: a meta-analysis update. *Mol Biol Rep, 39*(4), 3453-3460. doi: 10.1007/s11033-011-1117-3

Okada, Y., Mori, M., Yamada, R., Suzuki, A., Kobayashi, K., Kubo, M., . . . Yamamoto, K. (2008). SLC22A4 polymorphism and rheumatoid arthritis susceptibility: a replication study in a Japanese population and a metaanalysis. *J Rheumatol, 35*(9), 1723-1728.

Patsopoulos, N. A., & Ioannidis, J. P. (2010). Susceptibility variants for rheumatoid arthritis in the TRAF1-C5 and 6q23 loci: a meta-analysis. *Ann Rheum Dis, 69*(3), 561-566. doi: 10.1136/ard.2009.109447

Song b, G. G., Bae, S. C., Choi, S. J., Ji, J. D., & Lee, Y. H. (2012). Associations between interleukin-23 receptor polymorphisms and susceptibility to rheumatoid arthritis: a meta-analysis. *Mol Biol Rep, 39*(12), 10655-10663. doi: 10.1007/s11033-012-1955-7

Sugiyama, D., Nishimura, K., Tamaki, K., Tsuji, G., Nakazawa, T., Morinobu, A., & Kumagai, S. (2010). Impact of smoking as a risk factor for developing rheumatoid arthritis: a meta-analysis of observational studies. *Ann Rheum Dis, 69*(1), 70-81. doi: 10.1136/ard.2008.096487
