# Supplementary material for: Poor replication validity of biomedical association studies reported by newspapers
Source: PLoS One. 2017 Feb 21;12(2):e0172650. doi: 10.1371/journal.pone.0172650 (PMC5319681; doi:10.1371/journal.pone.0172650)
Supplement: S2 Text — (DOCX) [file pone.0172650.s002.docx]

Tables SI-S3 give the raw data shown in percentage in Figures 1-3. These tables and figures only take into account primary studies; meta-analyses articles were not considered.

Table S1: raw data of Figure 1

| **Study type** | number of primary studies in the database | | | | number of covered studies | | | |
| --- | --- | --- | --- | --- | --- | --- | --- | --- |
|  | all | 0< IF ≤ 10 | 10<IF<30 | IF ≥ 30 | all | 0< IF ≤ 10 | 10<IF<30 | IF ≥ 30 |
| **all studies** |  |  |  |  |  |  |  |  |
| initial | 405 | 287 | 72 | 46 | 53 | 8 | 15 | 30 |
| subsequent | 4318 | 3887 | 379 | 52 | 103 | 44 | 32 | 27 |
| all | 4723 | 4174 | 451 | 98 | 156 | 52 | 47 | 57 |
| **lifestyle** |  |  |  |  |  |  |  |  |
| initial | 39 | 30 | 5 | 4 | 5 | 1 | 1 | 3 |
| subsequent | 600 | 538 | 49 | 13 | 58 | 27 | 21 | 10 |
| all | 639 | 568 | 54 | 17 | 63 | 28 | 22 | 13 |
| **non-lifestyle** |  |  |  |  |  |  |  |  |
| initial | 366 | 257 | 67 | 42 | 48 | 7 | 14 | 27 |
| subsequent | 3718 | 3349 | 330 | 39 | 45 | 17 | 11 | 17 |
| all | 4084 | 3606 | 397 | 81 | 93 | 24 | 25 | 44 |

Table S2: raw data of Figure 2

| **Study type** | number of covered studies | | | | number of covered and confirmed | | | |
| --- | --- | --- | --- | --- | --- | --- | --- | --- |
|  | all | 0< IF ≤ 10 | 10<IF<30 | IF ≥ 30 | all | 0< IF ≤ 10 | 10<IF<30 | IF ≥ 30 |
| **all studies** |  |  |  |  |  |  |  |  |
| initial | 53 | 8 | 15 | 30 | 18 | 2 | 4 | 12 |
| subsequent | 103 | 44 | 32 | 27 | 58 | 20 | 17 | 21 |
| all | 156 | 52 | 47 | 57 | 76 | 22 | 21 | 33 |
| **lifestyle** |  |  |  |  |  |  |  |  |
| initial | 5 | 1 | 1 | 3 | 2 | 0 | 1 | 1 |
| subsequent | 58 | 27 | 21 | 10 | 29 | 14 | 9 | 6 |
| all | 63 | 28 | 22 | 13 | 31 | 14 | 10 | 7 |
| **non-lifestyle** |  |  |  |  |  |  |  |  |
| initial | 48 | 7 | 14 | 27 | 16 | 2 | 3 | 11 |
| subsequent | 45 | 17 | 11 | 17 | 29 | 6 | 8 | 15 |
| all | 93 | 24 | 25 | 44 | 45 | 8 | 11 | 26 |

Table S3: raw data of Figure 3

| Study type | number of studies covered | | | number of covered and confirmed | | |
| --- | --- | --- | --- | --- | --- | --- |
|  | PSY | NEURO | SOMA | PSY | NEURO | SOMA |
| initial | 23 | 11 | 19 | 5 | 4 | 9 |
| subsequent | 15 | 30 | 58 | 5 | 22 | 31 |
| all | 38 | 41 | 77 | 10 | 26 | 40 |

Subpopulation analysis of primary studies covered by three newspapers or more.

**Table S4:** Preferential coverage of initial findings and influence of the impact factor (IF).

| **Study type** | number of primary studies in the database | | | | number of covered studies | | | |
| --- | --- | --- | --- | --- | --- | --- | --- | --- |
|  | all | 0< IF ≤ 10 | 10<IF<30 | IF ≥ 30 | all | 0< IF ≤ 10 | 10<IF<30 | IF ≥ 30 |
| **all studies** |  |  |  |  |  |  |  |  |
| initial | 405 | 287 | 72 | 46 | 32 | 4 | 5 | 23 |
| subsequent | 4318 | 3887 | 379 | 52 | 70 | 23 | 23 | 24 |
| all | 4723 | 4174 | 451 | 98 | 102 | 27 | 28 | 47 |
| **lifestyle** |  |  |  |  |  |  |  |  |
| initial | 39 | 30 | 5 | 4 | 3 | 1 | 0 | 2 |
| subsequent | 600 | 538 | 49 | 13 | 42 | 15 | 17 | 10 |
| all | 639 | 568 | 54 | 17 | 45 | 16 | 17 | 12 |
| **non-lifestyle** |  |  |  |  |  |  |  |  |
| initial | 366 | 257 | 67 | 42 | 29 | 3 | 5 | 21 |
| subsequent | 3718 | 3349 | 330 | 39 | 28 | 8 | 6 | 14 |
| all | 4084 | 3606 | 397 | 81 | 57 | 11 | 11 | 35 |

**Table S5:** Replication validity of primary articles reported by three newspapers or more.

| **Study type** | number of covered studies | | | | number of covered and confirmed | | | |
| --- | --- | --- | --- | --- | --- | --- | --- | --- |
|  | all | 0< IF ≤ 10 | 10<IF<30 | IF ≥ 30 | all | 0< IF ≤ 10 | 10<IF<30 | IF ≥ 30 |
| **all studies** |  |  |  |  |  |  |  |  |
| initial | 32 | 4 | 5 | 23 | 13 | 1 | 1 | 11 |
| subsequent | 70 | 23 | 23 | 24 | 40 | 8 | 13 | 19 |
| all | 102 | 27 | 28 | 47 | 53 | 9 | 14 | 30 |
| **lifestyle** |  |  |  |  |  |  |  |  |
| initial | 3 | 1 | 0 | 2 | 1 | 0 | 0 | 1 |
| subsequent | 42 | 15 | 17 | 10 | 20 | 6 | 8 | 6 |
| all | 45 | 16 | 17 | 12 | 21 | 6 | 8 | 7 |
| **non-lifestyle** |  |  |  |  |  |  |  |  |
| initial | 29 | 3 | 5 | 21 | 12 | 1 | 1 | 10 |
| subsequent | 28 | 8 | 6 | 14 | 20 | 2 | 5 | 13 |
| all | 57 | 11 | 11 | 35 | 32 | 3 | 6 | 23 |

**Table S6:** Replication validity of primary studies reported by three newspapers or more in three biomedical domains.

| Study type | number of studies covered | | | number of covered and confirmed | | |
| --- | --- | --- | --- | --- | --- | --- |
|  | PSY | NEURO | SOMA | PSY | NEURO | SOMA |
| initial | 14 | 5 | 13 | 4 | 2 | 7 |
| subsequent | 9 | 20 | 41 | 3 | 14 | 23 |
| all | 23 | 25 | 54 | 7 | 16 | 30 |
